# Metabolic Outcomes in Bariatric/Metabolic Surgery Individuals: Impact of Metabolic Health Definition, Type of Surgery, and Follow-Up Duration—An Observational, Retrospective Study

**DOI:** 10.3390/metabo16010047

**Published:** 2026-01-05

**Authors:** Anna Pluemacher, Cláudia Camila Dias, Bárbara Peleteiro, Denise Pinheiro, Paula Freitas, Eduardo Lima, Alexandra Leitão, Elisabete Martins, Maria João Martins

**Affiliations:** 1 Faculty of Nutritional Sciences, University of Potsdam, 14476 Potsdam, Germany; 2 Unit of Biochemistry, Department of Biomedicine, Faculty of Medicine, University of Porto, 4200-319 Porto, Portugal; 3 Knowledge Management Unit, Faculty of Medicine, University of Porto, 4200-319 Porto, Portugal; 4 RISE-Health, Department of Community Medicine, Information and Health Decision Sciences (MEDCIDS), Faculty of Medicine, University of Porto, 4200-319 Porto, Portugal; 5 Centro de Epidemiologia Hospitalar, Unidade Local de Saúde São João, 4200-319 Porto, Portugal; 6 Departamento de Ciências da Saúde Pública e Forenses e Educação Médica, Faculdade de Medicina, Universidade do Porto, 4200-319 Porto, Portugal; 7 EPIUnit, Instituto de Saúde Pública, Universidade do Porto, 4050-600 Porto, Portugal; 8 Laboratório para a Investigação Integrativa e Translacional em Saúde Populacional (ITR), Universidade do Porto, 4050-600 Porto, Portugal; 9 Faculdade de Medicina, Universidade do Porto, 4200-319 Porto, Portugal; 10 Instituto de Investigação e Inovação em Saúde (i3S), Universidade do Porto, 4200-135 Porto, Portugal; 11 Integrated Responsibility Center for Obesity (CRI-O), São João Local Health Unit, 4200-319 Porto, Portugal; 12 Departamento de Medicina Interna, Unidade Local de Saúde de Barcelos/Esposende, 4754-909 Barcelos, Portugal; 13 RISE-Health, Faculty of Medicine, University of Porto, 4200-319 Porto, Portugal; 14 Serviço de Cardiologia, ULS S. João EPE, 4200-319 Porto, Portugal; 15 Department of Medicine, Faculty of Medicine, University of Porto, 4200-319 Porto, Portugal

**Keywords:** bariatric surgery, gastric band, Roux-en-Y gastric bypass, sleeve gastrectomy, metabolic health phenotypes, metabolically healthy phenotype, metabolically unhealthy phenotype, overweight, obesity

## Abstract

Background: There is no standardized definition for metabolic health. Overweight and obesity are often linked to metabolic dysfunction. Bariatric surgery promotes body weight loss and cardiometabolic health improvement. Objective: We aim to characterize metabolic health using distinct definitions and evaluate anthropometric and cardiometabolic features, both before and after different surgery procedures. Methods: We studied 3313 individuals from CRI-O [Porto, PT; BMI 39.56 (42.60; 46.20) kg/m^2^; 36 (43; 51) y; 82.7% women] who underwent Roux-en-Y gastric bypass (RYGB; 61.7%), sleeve gastrectomy (30.9%), or gastric band (7.5%) surgery. Anthropometric and cardiometabolic features were assessed at baseline and at yearly follow-ups, up to 4 years; the same for cardiometabolic dysfunction characterization using NCEP ATP III, Karelis, Meigs, Khan, Pluemacher, and Schulze definitions. Results: Baseline metabolic health classification and metabolically unhealthy phenotype (MUH) post-surgery prevalence decrease show substantial variability depending on the definition used. Unlike relative body weight loss, the altered metabolic feature number in MUH remains unchanged. Changes in MUH prevalence do not reflect body weight loss, nor does the variation in MUH percentage fully align with changes in altered metabolic features. Blood pressure, C-reactive protein, antihypertensive medication, and HOMA-IR are key contributors to baseline MUH. Post-surgical changes in body weight, lipid profile, and C-reactive protein vary by procedure. RYGB yields greater weight loss and more often improves cardiometabolic markers. However, post-operative metabolic phenotype is independent of surgery type. Conclusions: Metabolic health phenotypes pre- and post-surgery vary by definition, and the latter are not solely driven by weight loss or surgery type. In this cohort, RYGB shows the strongest beneficial impact.

## 1. Introduction

In 2022, according to the World Health Organization (WHO), one billion people were living with obesity and 43% of adults were classified as having overweight [body mass index (BMI) ≥ 30 kg/m^2^ or within the range 25.0–29.9 kg/m^2^, respectively, for adults (distinct cut-off values are published for Asian populations)] [1,2,3]. Not only body weight but also metabolic health are relevant and intertwined features when evaluating risk factors for non-communicable diseases, such as hypertension [4,5,6], dyslipidaemia [4,5], type 2 diabetes [1,2,4,5,6,7,8], and several cardiovascular [1,2,3,4,5,6,7,8,9,10,11], chronic kidney [2,6,12] and respiratory diseases [1,4,5,6,13,14], as well as some types of cancer [1,2,4,5,6,7,15,16,17].

Higher body weight values and/or a metabolically unhealthy phenotype (MUH) strongly contribute to morbidity and premature mortality [1,2,6]. Body weight loss is a prime goal in individuals with overweight and obesity, aiming to preserve or regain their metabolic health, to prevent or reduce disability and, consequently, to prolong healthy ageing and avoid premature death. Bariatric/metabolic surgery plays an important role in successful body weight loss and improvement of future metabolic health; gastric band (whose use has been declining), Roux-en-Y gastric bypass (RYGB), and sleeve gastrectomy may provide different results that can change over time during follow-up [3,5,7,18,19,20,21,22,23,24,25,26,27,28,29].

As reviewed by Brandão et al. 2020 [30], Tanriover et al. 2023 [6], and Schulze et al. 2024 [2], among others, there is a subset of individuals with overweight or obesity who are considered metabolically healthy although, over time [2,6,30], they will progress towards an MUH status.

To identify the metabolically healthy phenotype (MH) and MUH groups, and considering the differences in the characterization of the metabolic impairment, several distinct definitions of metabolic health have been proposed and used [reviewed in [2,6,30], among others]. Distinct sets of features (and sometimes different cut-off values) characterise these definitions, including, for example, waist circumference, type 2 diabetes, insulin sensitivity/resistance, lipid profile, blood pressure, and/or fibrinolytic and inflammatory profiles [2,6,30], which explains the wide range of percentage values reported for MH and MUH in the literature.

In this study, we used six definitions that were specifically chosen due to their widespread application in clinical practice and/or the distinct sets of features they include, which distinctly capture clinically relevant dimensions of metabolic health and allow us to describe the evolution of the corresponding metabolic health phenotypes in a bariatric/metabolic surgery setting. NCEP ATP III definition is a well-established and (still) widely used guideline for diagnosing metabolic syndrome [31], which includes waist circumference (WSC, as visceral adiposity is central to metabolic dysfunction), blood pressure, lipid profile, and glucose [31,32,33,34,35]. As insulin resistance and inflammation are strongly relevant and interrelated features in metabolic dysfunction progression, for example, in the development of hypertension and hyperlipidemia [31,32,36], definitions that incorporate these parameters in different combinations were also used. Karelis et al.’s definition is based on only two features, lipid profile and insulin-resistance status [37,38], whereas Meigs et al.’s definition relies solely on the latter [39]. Khan et al.’s definition, in addition to the traditional metabolic syndrome components (but excluding WSC), includes one inflammatory marker [40]. Glucose, insulin resistance, systolic blood pressure (SBP), and inflammation were selected for the definition created by us, whereas Schulze et al.’s definition takes into account type 2 diabetes prevalence and SBP [2]. These latter two definitions combine two of the most prevalent metabolic impairments [insulin resistance/type 2 diabetes (in 2021, 508 million people were living with type 2 diabetes, 52.2% of which was high BMI-associated) [41] and high blood pressure (estimated to affect 33% of adults aged 30–79 y in 2024) [42]], with inflammation also included in the definition created by us. Similar to Khan et al.’s definition [40], our own definition does not include any anthropometric parameter, while Schulze et al.’s definition includes the waist-to-hip ratio [2].

To our knowledge, this is the first time a large population of individuals with overweight or obesity who underwent bariatric/metabolic surgery had their baseline metabolic health phenotype determined using six distinct definitions, aiming to characterize and compare the corresponding MH/MUH distributions at baseline within the same population. Furthermore, this approach allowed us to simultaneously characterise the anthropometric and metabolic post-surgery outcomes according to the same definitions during the 4-year follow-up period—becoming our second aim. Finally, we examined the impact of the type of bariatric/metabolic surgery on the anthropometric and metabolic post-surgery outcomes over follow-up—this became our third aim.

Corresponding to these aims, our research questions are as follows: (a) Which metabolic health definition most accurately classifies candidates for bariatric/metabolic surgery? (b) Which definition best captures metabolic improvement after bariatric/metabolic surgery? (c) Which bariatric/metabolic surgical procedure leads to the greatest improvements in anthropometric and metabolic features? and (d) Do the metabolic parameters that show the greatest improvement after bariatric/metabolic surgery correspond more closely to any particular definition of metabolic health?

## 2. Methods

### 2.1. Population

We performed an observational, retrospective study based on the medical records of individuals who underwent bariatric surgery at the Integrated Responsibility Centre for Obesity (CRI-O), of the São João Local Health Unit, Porto, Portugal, which was approved by the Ethics Committee of this medical institution (project number: CE-241-23; approval date: 23 November 2023).

Individuals with a BMI lower than 25 kg/m^2^ or women and men presenting WSC values ≤ 88 cm or ≤ 102 cm (*n* = 3), respectively, at baseline, as well as individuals who underwent revisional surgery (*n* = 526), were excluded from the analysis.

We enrolled 3313 individuals (Supplementary Table S1) who underwent bariatric/metabolic surgery between January 2010 and September 2021: gastric band (*n* = 245), RYGB (*n* = 2038), sleeve gastrectomy (*n* = 1020) and other procedures (miscellaneous bariatric/metabolic surgery group) (*n* = 10). After bariatric/metabolic surgery (baseline; time 0), follow-up evaluations were conducted annually for up to four years, with the first follow-up taking place one year after the surgical procedure.

### 2.2. Metabolic Health Phenotype Characterization

Metabolic health phenotypes were identified according to six different metabolic health definitions: NCEP ATP III modified from 2001, 2002, and 2005 (*n* = 1791) [33,34,35], Karelis et al. 2004 (*n* = 1955) [37,38], Meigs et al. 2006 (*n* = 2079) [39], Khan et al. 2011 (*n* = 2035) [40], our own, Pluemacher et al. 2024 (*n* = 1278), and Schulze et al. 2024 (*n* = 1902) [2].

Table 1 discloses the anthropometric and metabolic features, and the corresponding cut-off values, included in each of the six abovementioned definitions, both at baseline and follow-up. The phrase “altered metabolic features” refers to metabolic parameters that are impaired and contribute to the MUH classification.

Because the six definitions differ with respect to whether and how anthropometric parameters are included, the body weight/WSC categories were aligned as follows (Table 1): (a) as Khan et al. [40] consider WSC a BMI covariate and their definition excludes any antropometric feature but is applied on BMI-categorised individuals, we categorised individuals according only to BMI and grouped together individuals with overweight or obesity for both Khan et al.’s definition [40] and our definition; (b) as in Karelis et al.’s [37,38] and Meigs et al.’s [39] definitions neither BMI nor WSC are part of the metabolic criteria, individuals with overweight, obesity, or elevated WSC were grouped together to maintain consistency with definitions including these anthropometric measures (Khan et al.’s [40], Pluemacher et al.’s, and NCEP ATP III definitions [33,34,35]); and (c) for NCEP ATP III [33,34,35] and Schulze et al.’s [2] definitions the antropometric feature included in the definition is used for individuals selection/categorization.

For metabolic health phenotype identification at each follow-up, in order to maintain consistency in the anthropometric parameters used and to avoid classifying the same individual into more than one group, individuals were divided as follows: normal weight (BMI < 25 kg/m^2^ and WSC ≤ 102 cm for men or 88 cm for women), overweight (BMI 25–29.9 kg/m^2^ and WSC ≤ 102 cm for men or 88 cm for women), obesity (BMI ≥ 30 kg/m^2^ or WSC > 102 cm for men or 88 cm for women), and overweight + obesity (BMI ≥ 25 kg/m^2^ or WSC > 102 cm for men or 88 cm for women) (Table 1). The adjustment of the number of criteria was performed as follows. In definitions where WSC is one of the criteria, this parameter was already altered in our study population at baseline. Consequently, the maximum number of allowed abnormalities to be classified as metabolically healthy was therefore reduced (e.g., in NCEP ATP III [33,34,35]) (Table 1). At baseline, participants were considered MH only if they met one or none of the remaining criteria. The same logic was applied to the overweight/obesity groups at follow-up. For the normal-weight group at follow-up, the permissible number of abnormalities remained two or fewer, since WSC was generally within the normal range and verified to be below the cut-off (Table 1).

As Karelis et al. [37,38] do not include BMI or WSC in their definition, no adjustment was made in the number of criteria at baseline or follow-up; the same for Meigs et al.’s [39] and Khan et al.’s [40] definitions (Table 1). For the Schulze et al.’s definition [2] as well, no adjustment was needed in the number of criteria for metabolic health classification at baseline or follow-up, but the waist-to-hip ratio was replaced with BMI or WSC categorization in the latter (Table 1). For all definitions at follow-up, there is uniformity in the anthropometric categorization of the individuals.

Homeostatic Model Assessment for Insulin Resistance (HOMA-IR) [44] cut-off values for both Meigs et al.’s [39] and Pluemacher et al.’s definitions were both population and time-point-specific (75th percentile among the individuals without diabetes, either at baseline or at each of the four follow-ups; Table 1). Since we used all individuals enrolled in the study (regardless of the definition) to calculate the required percentiles, the code was the same for both definitions (and consequently the values).

For each time point (baseline or any follow-up) and for each definition, analyses included only individuals with complete anthropometric and metabolic data required for that definition; participants with missing values for any required feature (within each definition) were excluded. The overall set of individuals included at baseline is described in detail in Supplementary Table S1. Supplementary Figure S1A,B show how many individuals were eligible, how many had each outcome available at each time point, and how this looks by procedure. For consistency (since only diastolic blood pressure, hip circumference, heart rate, and albumin show a normal distribution), all results are presented as medians with 25th percentile and 75th percentile values. Sample sizes for each weight group, depending on the definitions and evaluation time points, are provided in Supplementary Table S2. These sample sizes reflect both the number of individuals with complete data and those who were lost to follow-up at each time point.

### 2.3. Statistical Analysis

Only individuals with complete anthropometric and metabolic data for a given definition, at each time point (baseline or follow-up), were included in that specific analysis. Consequently, an individual could be included at one time point and excluded at another, for a given definition, depending on data availability.

Continuous variables are presented as mean ± standard deviation or median with minimum and maximum, depending on the data distribution. Categorical variables are expressed as absolute (*n*) and relative (%) frequencies. Crosstables were used to assess overlaps and the distribution of categorical variables. The normality of data distribution was assessed using the Shapiro–Wilk test and visual analysis of histograms. For within-group comparisons (e.g., baseline vs. follow-up), parametric tests (paired Student’s *t*-test) were applied, depending on normality assumptions. Between-group comparisons were conducted using unpaired Student’s *t*-tests or one-way ANOVA for continuous variables, depending on the number of groups, and chi-square tests for categorical variables. When the ANOVA test revealed differences between groups, the Tukey post hoc test was used to determine between which groups these differences existed. This test makes all possible comparisons between pairs of means, controlling for type I error (false positives). Logistic regression was applied to assess the relationship between metabolic health phenotype and the start of overweight or obesity or relative body weight loss, adjusting for age at surgery or age, gender, type of surgery, BMI at baseline, and calendar year of surgery, respectively. The odds ratio (OR) and 95% confidence intervals (95% CI) are presented. Given the presence of missing data, longitudinal analyses were performed using linear mixed-effects models, adjusted for age, gender, BMI at baseline, type of surgery, and calendar year of surgery. This approach allows the inclusion of incomplete observations under the missing-at-random assumption. A significance level of 0.05 was used for all tests. Statistical analyses were performed using Statistical Package for the Social Sciences (SPSS) (vers. 29, 2023).

## 3. Results

### 3.1. Age of Start of Excess Weight, Body Mass Index and Relative Body Weight Loss

The analysis of three overweight and obesity markers was made: age of start of excess weight [overweight and obesity (Table 2)], BMI (Supplementary Table S2) and relative body weight loss (Table 3).

**Figure 1:**
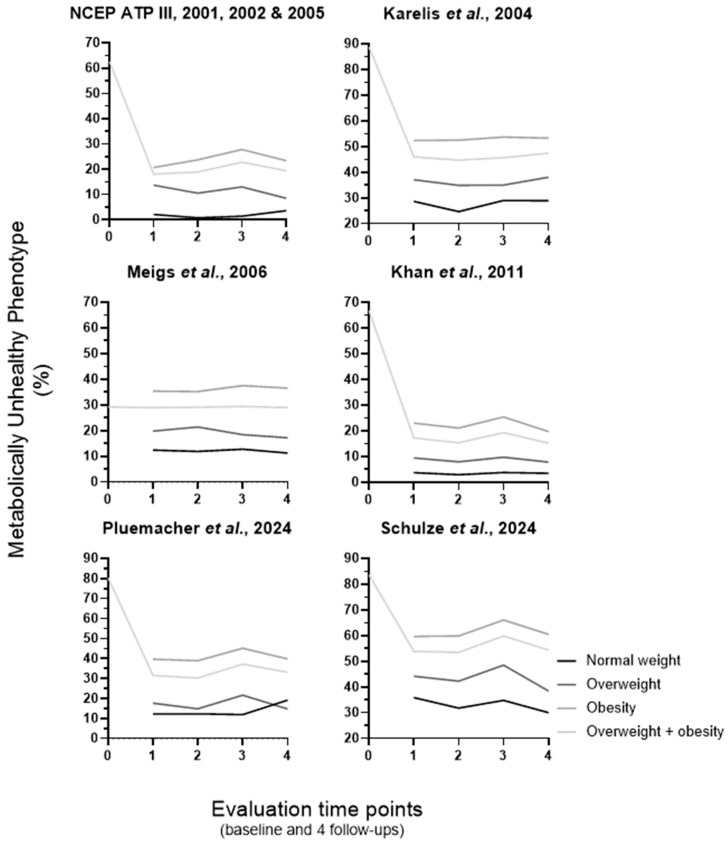
Metabolically unhealthy phenotype classification according to the six definitions used. At baseline, individuals were classified into the overweight and obesity group (BMI ≥ 25 kg/m^2^ or WSC above > 102 cm for men or 88 cm for women, respectively). For metabolic phenotype characterization over follow-up, individuals were divided into four body weight groups [normal weight (BMI < 25 Kg/m^2^ and WSC ≤ 102 cm for men or 88 cm for women), overweight (BMI 25–29.9 kg/m^2^ and WSC ≤ 102 cm for men or 88 cm for women), obesity (BMI ≥ 30 kg/m^2^ or WSC above > 102 cm for men or 88 cm for women) and overweight + obesity (BMI ≥ 25 kg/m^2^ or WSC above > 102 cm for men or 88 cm for women)]. NCEP ATP III modified from 2001, 2002, and 2005 (*n* = 1791) [33,34,35], Karelis et al. 2004 (*n* = 1955) [37,38], Meigs et al. 2006 (*n* = 2079) [39], Khan et al. 2011 (*n* = 2035) [40], our own, Pluemacher et al. 2024 (*n* = 1278) and Schulze et al. 2024 (*n* = 1902) [2] (sample size shown for baseline). BMI, body mass index; WSC, waist circumference. 0, baseline; 1 to 4, first to fourth follow-up (after bariatric/metabolic surgery, follow-up lasted up to four years (one follow-up per year, and the first follow-up was one year after the surgery).

The median (min; max) age at surgery was 39 (17; 67) y for individuals that started excess weight at childhood (<10 y), 38 (19; 66) y for those that started excess weight at adolescence (10–20 y), and 47 (22; 68) y for those that started excess weight when adults (>20 y). Independent of the metabolic health definition and baseline metabolic health phenotype, overweight and obesity were less frequently initiated during adolescence (15.0–21.6%); and regardless of the metabolic health definition, the percentage of MUH was consistently higher when overweight or obesity began in adulthood (48.2–51.4%). For all definitions but the Meigs et al.’s definition, and independent of the age of start of overweight or obesity, MH at baseline includes a lower percentage of individuals than MUH at baseline (Figure 1; Table 2). The percentage of MUH within each age group for the start of excess weight was significantly different for NCEP ATP III (*p* = 0.049), Karelis et al.’s (*p* = 0.006), Khan et al.’s (*p* = 0.024) and Schulze et al.’s (*p* < 0.001) definitions, being more prevalent in the adult age group (Table 2). Taking childhood onset of excess weight as the reference, the odds ratio for MUH is higher among individuals classified according to the Karelis et al.’s definition who first became overweight or obese in adulthood (Table 2).

At baseline, and without metabolic health characterization, median BMI ranged from 41.65 (38.79; 44.95) to 42.70 (39.75; 46.10) kg/m^2^ for individuals included in the Pluemacher et al.’s and Schulze et al.’s definitions, respectively. BMI decreased after bariatric/metabolic surgery for all definitions (at the first follow-up, one year after surgery); likewise, for all definitions, the decrease was smaller at the third and fourth follow-ups (three and four years after surgery; Supplementary Table S2).

For all definitions, and taking as reference the metabolic health phenotype group at baseline, the relative body weight loss at the first follow-up (from 31.30 ± 10.48 to 34.66 ± 8.83%) is significantly higher than at the third (28.98 ± 11.60 to 32.54 ± 9.96%) and fourth follow-ups [26.89 ± 11.74 to 30.32 ± 10.93%; *p*-values ranging from *p* < 0.05 to *p* < 0.001 for MH and *p* < 0.001 for MUH, the exception for MH according to both our own definition (the same pattern, although the difference did not reach statistical significance) and the Karelis et al.’s definition (a tendency was found at the third follow-up, *p* = 0.072)]. Independent of the metabolic health definition and baseline phenotype, relative body weight loss is similar in the first and second follow-ups, while always significantly smaller in the fourth vs. the third follow-ups. For both the third and fourth follow-ups, the smaller and higher relative body weight loss values were found for the NCEP ATP III and Khan et al. MH populations, respectively. Only for Khan et al.’s definition is there a statistically significant difference (*p* < 0.005), both at the first and second follow-ups, with a similar trend observed at the third follow-up (*p* = 0.096), when comparing the relative body weight loss between individuals classified as MH or MUH at baseline. Relative body weight loss is higher in individuals within the MH group at baseline (Table 3). Adjustment for age, gender, type of surgery, BMI at baseline, and calendar year of surgery reduced the intensity of the pattern for the Khan et al.’s definition (at the first and second follow-ups, the odds ratios are as follows: 0.987 [0.939–1.036], *p* = 0.083 and 0.985 [0.970–0.999], *p* = 0.046, respectively) and made the pattern evident for the Meigs et al.’s definition at the third and fourth follow-ups (0.970 [0.964–0.994], *p* = 0.008; 0.980 [0.962–0.999], *p* = 0.034). At the first follow-up, individuals included in the NCETP ATP III definition, within both metabolic health phenotypes, present the smallest relative body weight loss, while MH individuals included in the Khan et al.’s definition and MUH individuals included in the Pluemacher et al.’s definition show the highest relative body weight loss (MH: NCETP ATP III vs. Kan et al. *p* < 0.001; MUH: NCETP ATP III vs. Pluemacher et al. *p* < 0.001) (Table 3).

### 3.2. Metabolically Healthy and Unhealthy Phenotypes

As the main aim of bariatric/metabolic surgery is to reduce weight and improve metabolic health, only MUH was analyzed further.

The percentage of MUH according to each of the six definitions used for metabolic health classification, both at baseline and during follow-up, is disclosed in Figure 1. Only 199 individuals are simultaneously identified as MUH by all definitions, at baseline. At baseline, the highest MUH percentage occurs when using the Karelis et al.’s definition (88.8%), immediately followed by the Schulze et al.’s and our own definitions (83.7 and 80.4%, respectively); the smallest MUH percentage happens when the Meigs et al.’s definition is applied (29.2%), while intermediate values are disclosed for Khan et al.’s and NCEP ATP III definitions (66.8 and 62.6%, respectively). Khan et al.’s and NCEP ATP III definitions align most closely in identifying MH and MUH at baseline (κ = 0.781; 95% CI: 0.743–0.819), but the remaining definitions do not identify the same individuals [kappa values range from κ = 0.009, 95% CI: 0.029–0.150 (Pluemacher et al.’s vs. Karelis et al.’s definition) to κ = 0.506, 95% CI: 0.451–0.556 (Pluemacher et al.’s vs. Khan et al.’s definition)].

The drop in MUH percentage from baseline to the first follow-up (considering the overweight + obesity group for both time points) is 49.6, 48.9, 44.5, 42.8, and 29.8% for Khan et al.’s, Pluemacher et al.’s, NCEP ATP III, Karelis et al.’s, and Schulze et al.’s definitions, respectively (being negligible for the Meigs et al.’s definition: 0.3%). For all six definitions, during follow-up, the order of MUH percentage is normal weight < overweight < overweight + obesity < obesity groups (one exception occurred with our own definition: at the fourth follow-up, the order is reversed for the normal weight and overweight groups). Along the follow-up period, the pattern of MUH percentage variation is similar, for overweight, obesity, and overweight + obesity groups, for NCEP ATP III, Khan et al.’s, Pluemacher et al.’s, and Schulze et al.’s definitions: an increase from the first to the third follow-up (the highest MUH percentage value for the Schulze et al.’s definition: 66.1%) and then a decrease in the fourth follow-up (the smallest MUH percentage value for the Khan et al.’s definition: 7.8%). In contrast, the Meigs et al.’s definition demonstrated a relatively stable percentage of MUH across all time points and within each of the four weight groups (11.3–37.5%; Figure 1).

**Figure 2:**
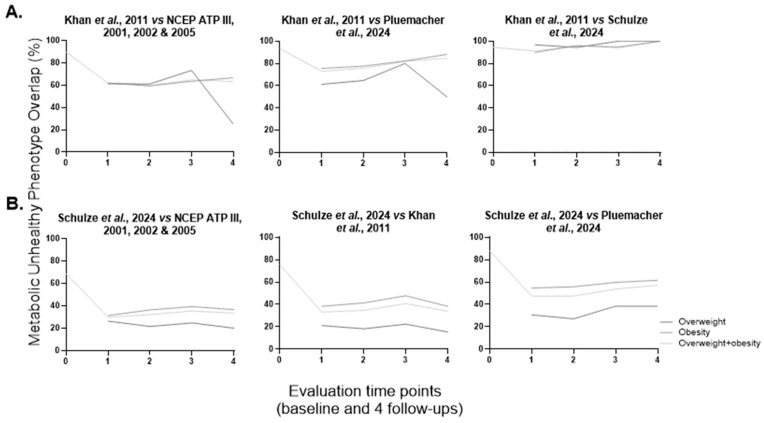
Overlap of populations with a metabolically unhealthy phenotype. (**A**). Khan et al.’s vs. NCEP ATP III, Pluemacher et al.’s, and Schulze et al.’s definitions; (**B**). Schulze et al.’s vs. NCEP ATP III, Khan et al.’s, and Pluemacher et al.’s definitions. At baseline, individuals were classified into the overweight and obesity group (BMI ≥ 25 kg/m^2^ or WSC above > 102 cm for men or 88 cm for women). For metabolic phenotype characterization over follow-up, individuals were divided into four body weight groups [normal weight (BMI < 25 kg/m^2^ and WSC ≤ 102 cm for men or 88 cm for women), overweight (BMI 25–29.9 kg/m^2^ and WSC ≤ 102 cm for men or 88 cm for women), obesity (BMI ≥ 30 kg/m^2^ or WSC above > 102 cm for men or 88 cm for women), and overweight + obesity (BMI ≥ 25 kg/m^2^ or WSC above > 102 cm for men or 88 cm for women)]. At baseline, *n* = 1791 for NCEP ATP III modified from 2001, 2002, and 2005 [33,34,35], *n* = 2035 for Khan et al. 2011 [40], *n* = 1278 for Pluemacher et al. 2024, and *n* = 1902 for Schulze et al. 2024 [2]. BMI, body mass index; WSC, waist circumference. 0, baseline; 1 to 4, first to fourth follow-up (after bariatric/metabolic surgery, follow-up lasted up to four years (one follow-up per year, and the first follow-up was one year after the surgery).

Aiming to investigate whether the overlap in the MUH population could explain the similar temporal pattern of MUH percentage, as just described for the NCEP ATP III, Khan et al.’s, Pluemacher et al.’s, and Schulze et al.’s definitions, the degrees of overlap between the Khan et al. MUH population against the NCEP ATP III, Pluemacher et al. or Schulze et al. MUH populations were calculated (Figure 2A). The baseline Khan et al. sample size (*n* = 2035, the largest among these four metabolic health definitions) determined the direction of the overlapping analysis. At baseline, the overlap values are 89.8, 93.4 and 94.5%, respectively, for NCEP ATP III, Pluemacher et al.’s, and Schulze et al.’s definitions. There is an almost complete and stable overlap between Khan et al. and Schulze et al. MUH populations across all evaluation time points and weight groups (89.9–100%). At the first follow-up, for the overweight + obesity group, there is a drop in overlap values against the NCEP ATP III and Pluemacher et al. MUH individuals (27.9 and 20.4%, respectively); unexpectedly (given the aforementioned similar pattern of the variation of MUH percentage), in a similar pattern for the NCEP ATP III and Pluemacher et al.’s definitions, overlap values vary from 25.0 to 73.3% and 50.0 to 88.1%, respectively, over the follow-up and across the three weight groups (Figure 2A). As one would anticipate from the distinct temporal patterns of the Khan et al. and Meigs et al. MUH percentage trajectories (Figure 1), the overlap values between Khan et al. and Meigs et al. MUH populations are not as high as the ones for Khan et al. vs. NCEP ATP III, Pluemacher et al., and Schulze et al. MUH populations (38% at baseline, and 33.3 to 71.3% during follow-up and across the three weight groups).

Unlike the definition by Meigs et al., and unexpectedly given the variation in MUH percentages over time, the overlap is higher for the MUH populations defined by Khan et al. and Karelis et al. (94.7% at baseline; 57.1 to 74.3% over the follow-up and across the three weight groups). Subsequently, we examined the overlap of the Schulze et al. MUH population (the definition presenting the most evident abovementioned pattern of MUH percentage variation along time) and the NCEP ATP III, Khan et al., and Pluemacher et al. MUH populations (Figure 2B). At baseline, the overlap values are 68.8, 75.5 and 87.8%, respectively (Figure 2B), and there is no (almost)complete overlap as seen before for Khan et al. and Schulze et al. MUH populations throughout the follow-up period and across the three weight groups (Figure 2A,B). Over the follow-up and across the three weight groups, the values ranged from 20.0 to 39.4%, 15.4 to 47.8% and 27.2 to 61.6%, for NCEP ATP III, Khan et al., and Pluemacher et al. MUH populations, respectively. The drop of the overlap of MUH, at the first follow-up, within the overweight + obesity group, was 39.0, 42.4 and 40.4%, respectively, for NCEP ATP III, Khan et al., and Pluemacher et al. MUH populations (Figure 2B). Overlap of MUH according to Schulze et al.’s vs. Meigs et al.’s definitions does not change over time for any of the three weight groups (21.8–45.4%; baseline 31.9%), while for the overlap of MUH according to Schulze et al.’s vs. Karelis et al.’s definitions, the results are similar to Schulze et al. vs. Pluemacher et al. MUH populations (88.3% at baseline and a drop of 38.6% at the first follow-up for the overweight + obesity group; 36.8–59.9% during follow-up and across the three weight groups).

**Figure 3:**
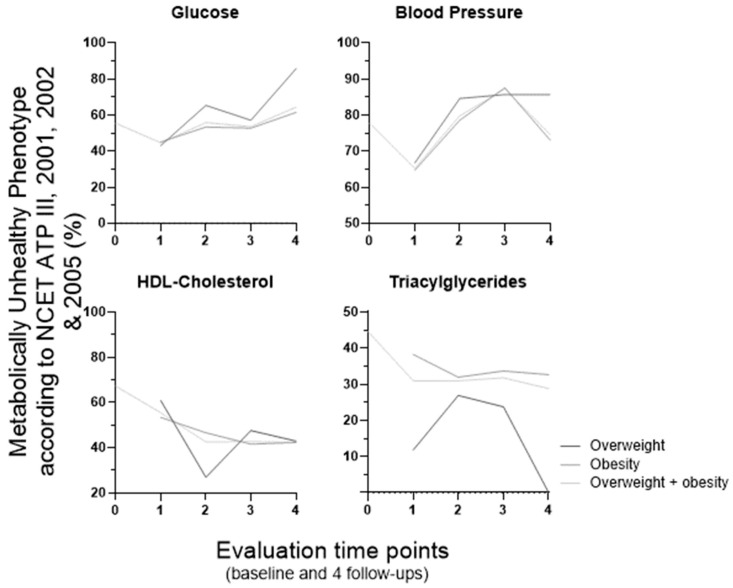
Percentage of MUH individuals with altered metabolic features according to the NCEP ATP III definition, modified from 2001, 2002, and 2005 [33,34,35] (baseline: *n* = 1791). At baseline, individuals were classified into the overweight and obesity group (BMI ≥ 25 kg/m^2^ or WSC above > 102 cm for men or 88 cm for women). For metabolic phenotype characterization over follow-up, individuals were divided into four body weight groups [normal weight (BMI < 25 kg/m^2^ and WSC ≤ 102 cm for men or 88 cm for women), overweight (BMI 25–29.9 kg/m^2^ and WSC ≤ 102 cm for men or 88 cm for women), obesity (BMI ≥ 30 kg/m^2^ or WSC above > 102 cm for men or 88 cm for women) and overweight + obesity (BMI ≥ 25 kg/m^2^ or WSC above > 102 cm for men or 88 cm for women)]. BMI, body mass index; MUH, metabolically unhealthy phenotype; WSC, waist circumference. 0, baseline; 1 to 4, first to fourth follow-up (after bariatric/metabolic surgery, follow-up lasted up to four years (one follow-up per year, and the first follow-up was one year after the surgery).

**Figure 4:**
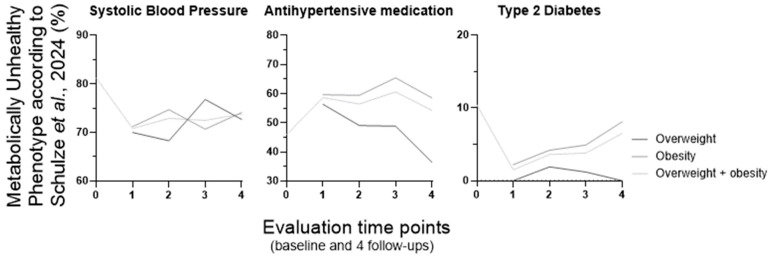
Percentage of MUH individuals with altered metabolic features according to the Schulze et al.’s 2024 [2] definition (baseline: *n* = 1902). At baseline, individuals were classified into the overweight and obesity group (BMI ≥ 25 kg/m^2^ or WSC above > 102 cm for men or 88 cm for women). For metabolic phenotype characterization over follow-up, individuals were divided into four body weight groups [normal weight (BMI < 25 kg/m^2^ and WSC ≤ 102 cm for men or 88 cm for women), overweight (BMI 25–29.9 kg/m^2^ and WSC ≤ 102 cm for men or 88 cm for women), obesity (BMI ≥ 30 kg/m^2^ or WSC above > 102 cm for men or 88 cm for women) and overweight + obesity (BMI ≥ 25 kg/m^2^ or WSC above > 102 cm for men or 88 cm for women)]. BMI, body mass index; MUH, metabolically unhealthy phenotype; WSC, waist circumference. 0, baseline; 1 to 4, first to fourth follow-up (after bariatric/metabolic surgery, follow-up lasted up to four years (one follow-up per year, and the first follow-up was one year after the surgery).

**Figure 5:**
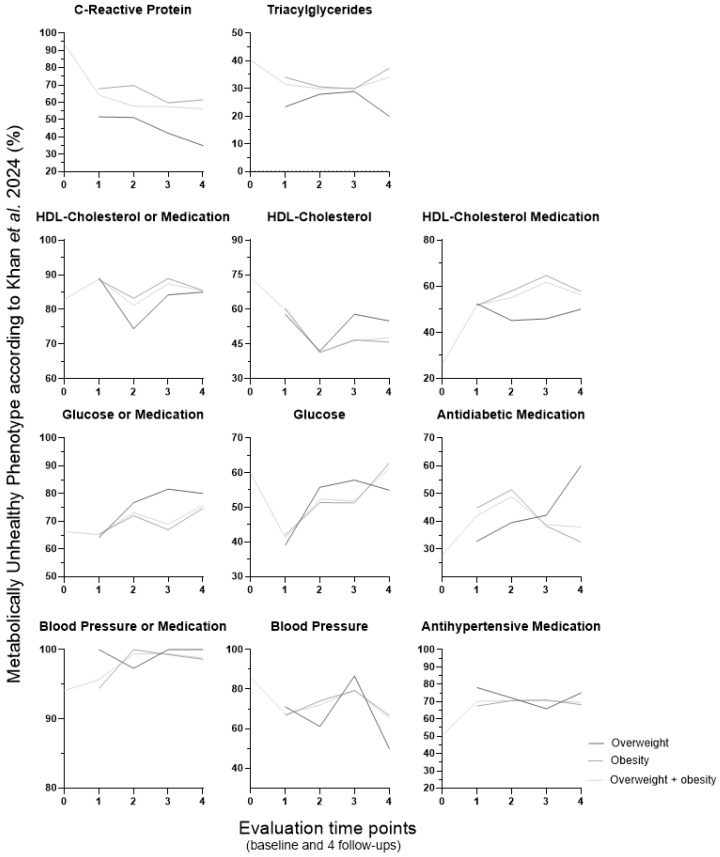
Percentage of MUH individuals with altered metabolic features according to the Khan et al.’s 2011 [40] definition (baseline: *n* = 2035). At baseline, individuals were classified into the overweight and obesity group (BMI ≥ 25 kg/m^2^ or WSC above > 102 cm for men or 88 cm for women). For metabolic phenotype characterization over follow-up, individuals were divided into four body weight groups [normal weight (BMI < 25 kg/m^2^ and WSC ≤ 102 cm for men or 88 cm for women), overweight (BMI 25–29.9 kg/m^2^ and WSC ≤ 102 cm for men or 88 cm for women), obesity (BMI ≥ 30 kg/m^2^ or WSC above > 102 cm for men or 88 cm for women) and overweight + obesity (BMI ≥ 25 kg/m^2^ or WSC above > 102 cm for men or 88 cm for women)]. BMI, body mass index; MUH, metabolically unhealthy phenotype; WSC, waist circumference. 0, baseline; 1 to 4, first to fourth follow-up (after bariatric/metabolic surgery, follow-up lasted up to four years (one follow-up per year, and the first follow-up was one year after the surgery).

**Figure 6:**
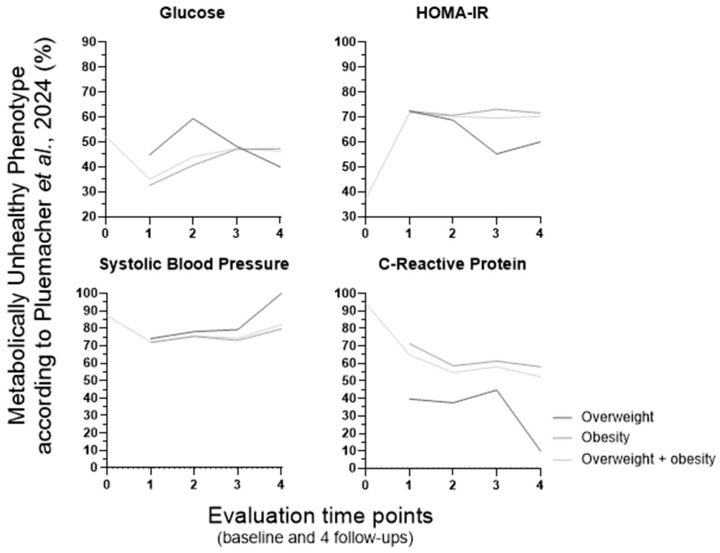
Percentage of MUH individuals with altered metabolic features according to the Pluemacher et al.’s 2024 definition (baseline: *n* = 1278). At baseline, individuals were classified into the overweight and obesity group (BMI 30 ≥ kg/m^2^ or WSC above > 102 cm for men or 88 cm for women). For follow-up metabolic phenotype characterization, individuals were divided into four body weight groups [normal weight (BMI < 25 kg/m^2^), overweight (BMI 25–29.9 kg/m^2^), obesity (BMI 30 ≥ kg/m^2^ or WSC above > 102 cm for men or 88 cm for women) and overweight + obesity (BMI 25 ≥ kg/m^2^ or WSC above > 102 cm for men or 88 cm for women)]. BMI, body mass index; HOMA-IR, Homeostatic Model Assessment for Insulin Resistance; MUH, metabolically unhealthy phenotype; WSC, waist circumference. WSC, waist circumference. 0, baseline; 1 to 4, first to fourth follow-up (after bariatric/metabolic surgery, follow-up lasted up to four years (one follow-up per year, and the first follow-up was one year after the surgery).

To investigate whether specific features contribute to the observed variation in MUH percentage over time, we examined the altered metabolic features associated with MUH (expressed as the percentage of MUH individuals presenting each impaired metabolic parameter) according to the NCEP ATP III, Schulze et al.’s, Khan et al.’s, and our own definitions (Figure 3, Figure 4, Figure 5 and Figure 6, respectively). These data are presented for the overweight, obese, and combined overweight + obese groups, across all time points.

Over the course of the entire experimental period and across the three weight groups, the percentage of MUH individuals with altered metabolic features varied in different ways, making it challenging to fulfill our goal. But these four definitions share one feature, which is high blood pressure (and/or a blood pressure-related parameter, namely the use of antihypertensive medication). Longitudinal percentage changes in hypertension prevalence and antihypertensive therapy use seem to contribute to that pattern of MUH percentage variation over time in NCEP ATP III (in obesity and overweight + obesity groups; Figure 3) and Schulze et al. (also in obesity and overweight + obesity groups; Figure 4) populations, respectively. When analyzed separately, high blood pressure, but not the use of antihypertensive medication, appears to contribute to that variation pattern of MUH in the Khan et al.’s definition (in all three weight groups). Although these two features are not considered separately in the Khan et al.’s definition, they are presented individually within Figure 5. Furthermore, low HDL–cholesterol and/or the use of medication for HDL–cholesterol management also contribute to that variation pattern (but differently, when considering the weight groups; Figure 5). However, in the Pluemacher et al.’s definition, variation of the percentage of MUH individuals with high C-reactive protein, but not of those with high systolic blood pressure, seems to be responsible for the abovementioned MUH variation pattern (in all three weight groups; Figure 6).

Overall, the proportion of MUH individuals with high C-reactive protein levels decreases across all weight groups in the Khan et al.’s definition (Figure 5). In the Schulze et al.’s definition, type 2 diabetes prevalence increases consistently from the first to the fourth follow-up in the obesity and overweight + obesity groups (Figure 4). In all three weight groups, (a) overall, the evolution of the percentage of MUH individuals with high triacylglycerides, unlike that of those with low HDL–cholesterol, is similar for NCEP ATP III (Figure 3), Karelis et al.’s, and Khan et al.’s (Figure 5) definitions, and (b) the percentage of MUH individuals with low HDL–cholesterol decreases similarly for NCEP ATP III (Figure 3), Karelis et al.’s, and Khan et al.’s (Figure 5) definitions only up to the second follow-up.

At baseline, the parameters driving MUH phenotype identification vary across the definitions. Blood pressure is the most frequently decisive parameter when using NCEP ATP III (77.7% of MUH individuals have elevated blood pressure; Figure 3), Schulze et al. (81.2% display elevated systolic blood; Figure 4) and Khan et al. [94.1% reveal elevated blood pressure and/or use of antihypertensive medication (85.8% have elevated blood pressure); Figure 5] definitions. In the latter definition, C-reactive protein is also quite relevant (elevated in 93.7% of MUH individuals). In the Pluemacher et al.’s definition (Figure 6), C-reactive protein is the most decisive parameter, followed closely by systolic blood pressure (being elevated in 94.2% and 87.2% of MUH individuals, respectively). These findings suggest that blood pressure, use of antihypertensive medication, and C-reactive protein, when considered as parameters for metabolic health characterization, frequently contribute to baseline MUH, strengthening and supporting the aforementioned relevance of the variation of high blood pressure, use of antihypertensive medication and high C-reactive protein percentages in MUH percentage variation over time (Figure 1). By contrast, in the Karelis et al.’s definition, high HOMA-IR is the dominant factor for baseline MUH phenotype identification (89.7%).

Given that definitions of metabolic health differ in the nature and the number of features they consider, we also examined whether the number of altered metabolic features that determines the MUH classification changes with the surgery procedure (baseline vs. first follow-up) and throughout the follow-up period. In Table 4, the number of altered metabolic features in MUH can be seen for five of the definitions used (the Meigs et al.’s definition was excluded because it includes just one metabolic parameter).

Within the overweight + obesity group, from baseline to the first follow-up, there is a significant drop in the number of altered metabolic features for all five definitions (14.06, 17.53, 8.67, 9.96, and 26.82% for NCEP ATP III, Karelis et al.’s, Khan et al.’s, our own, and Schulze et al.’s definitions, respectively; *p* < 0.05; Table 4); also, when considering the other three follow-ups, for the same weight group, the drop vs. baseline was statistically significant (*p* < 0.05; Table 4). The majority of the altered metabolic features included in the five definitions just mentioned have their percentages decreased from baseline to the first follow-up; those that do not have the percentage decreased (and in fact have it increased) are high total and high LDL–cholesterol in the Karelis et al.’s definition, use of antihypertensive medication in the Schulze et al.’s (Figure 4) and Khan et al.’s (Figure 5) definitions, use of medication for diabetes and HDL–cholesterol management in the Khan et al.’s definition (Figure 5), as well as high HOMA-IR in the Pluemacher et al.’s definition (Figure 6). Since Meigs et al. have a population-specific cut-off for HOMA-IR, the % drop in MUH is only very small, even though HOMA-IR values decrease 3.1 times from baseline to the first follow-up. For each definition and weight group, no significant differences were observed for the number of altered metabolic features in MUH across the different follow-ups (Table 4), unlike for relative body weight loss across the different follow-ups, when considering the metabolic health phenotype of each definition at baseline (Table 3).

### 3.3. Distribution of the Type of Surgery According to the Metabolic Health Phenotypes

For all analyses related to the type of surgery, only RYGB, sleeve gastrectomy and gastric band procedures were included, as other types of surgeries (miscellaneous bariatric/metabolic surgery group), owing to the small sample size, were excluded.

For MH and MUH at baseline, in all definitions, the most frequently performed surgery is RYGB (62.6–65.0%), and the least performed surgery is gastric band (0.5–13.6%). Within the MH and MUH individuals selected by Khan et al.’s and our own definition, a gastric band was performed in less than 1% of them. At baseline, the distribution of RYGB, sleeve gastrectomy, and gastric band procedures showed no significant differences between MH and MUH, except for the individuals included in the NCEP ATP III definition (*p* < 0.005; Supplementary Table S3).

### 3.4. Type of Surgery vs. Relative Body Weight Loss and Metabolic Parameters (from the Six Metabolic Health Definitions)

The type of surgery significantly impacts relative body weight loss during the follow-up period (*p* < 0.001 at all follow-ups): higher relative body weight loss comparing RYGB to sleeve gastrectomy (2.53–4.78%), RYGB to gastric band (15.59–18.76%), and sleeve gastrectomy to gastric band (10.81–15.35%) (Table 5).

To further investigate whether the type of surgical intervention influenced the evolution of the metabolic parameters used in the six definitions over time, as well as relative body weight loss, changes in these parameters across the different surgical groups were evaluated using linear mixed-effects models. Supplementary Table S4 presents their absolute change between baseline (first follow-up for relative body weight loss) and the last follow-up, adjusted for age, gender, BMI at baseline, type of surgery, and calendar year of surgery. No statistically significant impact of the type of surgery upon (systolic and diastolic) blood pressure, glycated hemoglobin, HOMA-IR, and glucose occurs (Supplementary Table S4).

At the end of the follow-up, triglycerides and C-reactive protein are significantly higher (*p* < 0.001), and HDL–cholesterol is significantly lower (*p* < 0.026 and *p* < 0.001, respectively) in the gastric band compared to RYGB and sleeve gastrectomy (Supplementary Table S4). Total and LDL–cholesterol varied similarly: significantly higher in gastric band compared to RYGB and sleeve gastrectomy, and significantly higher in sleeve gastrectomy compared to RYGB (*p* < 0.001; Supplementary Table S4). Relative body weight loss is significantly lower in the gastric band compared to RYGB and sleeve gastrectomy, and significantly lower in sleeve gastrectomy compared to RYGB (*p* < 0.001; Supplementary Table S4).

Table 6 and Table 7 show diabetes and hypertension progression by surgery type, respectively.

Overall, across all surgery types, almost all individuals without diabetes at a given time point remained non-diabetic at the subsequent time point, with only up to 4% of them changing status, in a time- and surgery type-dependent manner, demonstrating high stability of non-diabetic status over time. Among participants with diabetes at baseline, more than two-thirds were non-diabetic at the first follow-up, with remission rates varying by surgery type and being highest for RYGB (95%) and lowest for gastric band (67%). At subsequent follow-ups, remission rates generally decreased (≤50%), also in a surgery type-dependent manner, with the exception of the transition from the second to the third follow-up for RYGB (67%) (Table 6).

Overall, across all surgery types, most individuals without high blood pressure at a given time point remained normotensive at the subsequent assessment, although with considerably more variation than observed for diabetes, and with transitions to hypertension ranging from 17% to 48%, in a time- and surgery type-dependent manner. Among participants with high blood pressure at baseline, remission at the first follow-up occurred in roughly half of the cases, with rates again varying by surgery type and being highest for RYGB (61%) and lowest for gastric band (44%). At the subsequent follow-ups, remission rates fluctuated and generally declined (≤56%) (Table 7).

Lastly, additionally, whether the metabolic health phenotype, at follow-ups one and two (only in OW + OB group), differed depending on the type of surgery performed was evaluated (Supplementary Table S5). This approach was chosen because most individuals remained overweight or obese during the follow-up period. By focusing on this group, the analysis included the largest possible sample size while maintaining clarity and avoiding overly complex data presentation. Follow-ups three and four were excluded from this analysis because relative body weight loss decreased (Table 3) and BMI increased (Supplementary Table S2) in these two last time points. No statistically significant differences occur between the first and second follow-up, in any definition, within each surgery group (Supplementary Table S5).

## 4. Discussion

To our knowledge, this is the first time individuals with overweight and obesity who underwent bariatric/metabolic surgery have their baseline metabolic health phenotype simultaneously determined by six distinct definitions [NCEP ATP III modified from 2001, 2002, and 2005 [33,34,35], Karelis et al. 2004 [37,38], Meigs et al. 2006 [39], Khan et al. 2011 [40], our own, Pluemacher et al. 2024, and Schulze et al. 2024 [2]]. Moreover, this is the first study to analyze anthropometric and metabolic post-surgery outcomes according to six distinct definitions and the type of bariatric/metabolic procedure.

The strengths of our observational, retrospective study include a large cohort of overweight and obese individuals of both sexes undergoing three different types of bariatric surgery (gastric band, sleeve gastrectomy, and RYGB), a longitudinal design with a follow-up period of 4 y, a comprehensive metabolic health assessment based on distinct definitions, and centralized laboratory analyses to ensure consistency in metabolic profiling. Some results from this article were presented orally at three conferences [45,46,47].

Metabolic health classification at baseline shows substantial variability depending on the definition used, ranging from 29.2 to 88.8% (Meigs et al.’s [39] and Karelis et al.’s [37,38] definitions, respectively) for MUH. This aligns with findings from previous studies reporting a wide range in the prevalence of metabolic health phenotypes, depending on the definition and population [2,21,30,48,49]. In studies that have specifically evaluated the prevalence of metabolic health phenotypes in populations referred for or undergoing bariatric/metabolic surgery, MUH ranges from 62.7 to 100%, depending on the stringency of the definition used, as well as population age and gender [19,20,21,24,26,27,28,50,51,52,53,54]. However, there is a limited number of studies focusing on metabolic health phenotypes evolution/improvement, or MUH conversion into MH, after bariatric/metabolic surgery [18,19,20,21,22,24,25,26,27,28]. This variability is also evident in our study, in the overlap of MUH individuals identified by the different definitions, which depends on the following: (a) target population, (b) specific criteria used to define metabolic health, and (c) direction of the overlap analysis [2]. In fact, only 6% of all individuals were simultaneously classified as metabolically unhealthy by the six definitions.

The primary goal of treating overweight and obesity is to improve metabolic health through excess body weight loss, with bariatric/metabolic surgery being a relevant approach [3,5,7,18,19,20,21,22,23,24,25,26,27,28].

In our study, MUH prevalence decreases, as expected, from baseline to first follow-up (1 y post-surgery; overweight + obese group), although the magnitude of this reduction varies across definitions: it is lowest among individuals classified as MUH by the Schulze et al.’s definition [2] and highest among those classified as MUH by the Khan et al.’s definition [40], with drops of 29.8 and a 49.6%, respectively. The exception is the Meigs et al.’s definition [39], owing to the variability of the HOMA-IR cut-off value. Post-surgery body weight changes seem to play a significant role in the metabolic health classification during the follow-up period, as the highest prevalence of MUH is consistently observed in the obese group, followed by the overweight + obese group. This finding is consistent with the well-established association between a higher body weight/BMI and an increased risk for metabolic abnormalities [1,2,3,4,5,6,7,8,9,10,11].

An overall relative body weight loss at the first follow-up of approximately one-third of the initial body weight is disclosed, which remains stable at the second follow-up (2 y post-surgery), decreases at the third, and further declines at the fourth (3 and 4 y post-surgery, respectively), for both phenotypes. Although body weight and BMI are commonly associated with metabolic dysfunction [1,2,4,5,6,7,8], here, the number of altered metabolic features in MUH does not significantly change during follow-up, in contrast with the just described weight loss. Moreover, the trajectory of the overall relative body weight loss in MUH individuals does not parallel changes in the proportion of MUH cases, nor does the variation pattern of MUH percentage fully aligns with the variation pattern of the number of altered metabolic features in MUH. In line with these apparent inconsistencies, Storms et al. [24] reported that relapse to MUH, after bariatric/metabolic surgery-induced conversion of MUH into MH, is not linked to body weight regain and highlighted that the MH condition remains unstable after body weight loss (and so, consequently, the same applies for MUH), even when body weight is maintained. Also, in accordance, Weiss et al. [18] and Luna et al. [27] identified initial fat distribution (visceral vs. subcutaneous), rather than pre-surgical BMI or bariatric surgery-induced weight loss, as the key determinant of metabolic improvement. Similarly, as observed by Lee et al. [22], MUH-to-MH conversion was not associated with a reduction of BMI after surgery. Instead, weight reduction induced specific abdominal fat depot changes (namely, a reduction in retroperitoneal adipose tissue volume and an improvement in intraperitoneal adipose tissue quality) that were positively associated with metabolic health conversion.

The aforementioned apparent inconsistencies may also be further explained by the increased production of gut hormones after bariatric/metabolic surgery, which improves metabolic health independently of body weight loss. In fact, RYGB, known to substantially and significantly increase glucagon-like peptide-1 (GLP-1) secretion [28,55,56], is the most prevalent surgery type in our study (61.7%). Age may also be a contributing factor, as Weiss et al. [18] observed that individuals who no longer met the criteria for metabolic syndrome six months following bariatric surgery (including RYGB, laparoscopic gastric band, sleeve gastrectomy, and duodenal switch procedures) were significantly younger than those who retained the diagnosis. Notably, both groups exhibited comparable outcomes in terms of weight, excess weight loss, BMI, and fat mass [18]. Furthermore, the differential trajectory of the proportion of altered metabolic features and the use of medication (which affects the proportion of impaired features beyond body weight dynamics) found by us further contributes to the explanation. It is also important to highlight that, in our study, the onset of excess body weight or obesity during adulthood is associated with MUH. Although this association is not consistently reported in the literature [57,58,59], discrepancies may be explained by differences in population characteristics, including gender distribution, BMI ranges, and age cut-offs used to define the onset of excess weight or obesity.

Goday et al. [26] observed a consistent pattern of an increase in BMI change and body weight loss (total and excess) up to 1 y, stability up to 1.5–2 y and then a decrease up to 5 y after bariatric surgery [by enrolling 191 individuals: BMI > 40 kg/m^2^, 83.8% female, 78.5% classified as MUH (according to Wildman et al. [60])]–an overall pattern similar to ours. In Goday’s study, 1 y after bariatric surgery, MH individuals started showing higher BMI change and excess weight loss vs. MUH individuals (more pronounced at 4 y; *p* = 0.082 and *p* = 0.036, respectively). These anthropometric differences were associated with individuals’ age [as MH subjects were younger (42.4 ± 11.0 y for MH vs. 46.3 ± 7.9 y for MUH)], but the impact of surgery type (no statistical difference in RYGB and sleeve gastrectomy distribution between MH and MUH) or gender (tendency for a higher female percentage in MH) was not discussed [19,26]. Accordingly, Pelascini et al. [20] described that MH [including individuals who were mostly women (90.5%) and who were significantly younger than MUH individuals] was significantly linked to a higher proportion of excess BMI loss regardless of age, gender, or revision surgeries, with weight loss stabilizing in both phenotypes between 12 months and 2 y post-surgery [102 individuals divided into MH and MUH, according to Wildman criteria, completed the 2-y follow-up after RYGB (43% male; 45.6 ± 10 y; 45 ± 6.6 kg/m^2^) [60]].

In our study, the pattern observed in relative body weight loss between MH and MUH up to the second follow-up (higher in MH), seen only when using the Khan et al.’s definition [40], loses relevance after adjustment for age, gender, type of surgery, BMI at baseline, and calendar year of surgery. Curiously, (a) within definitions by Schulze et al. [2] (used as an example) and Khan et al. [40], MUH individuals are significantly older than the MH individuals, but within the Schulze et al.’s definition [2] the relative body weight loss is similar for both metabolic health phenotypes, and (b) no significant differences are observed in MH, whereas significant differences in gender distribution (*p* = 0.012) are found in MUH between Schulze et al. [2] (MUH: 17.5% men vs. 82.5% women) and Khan et al. [40] (MUH: 22.4% men vs. 77.6% women) definitions. Lee et al. disclosed that MH individuals were more likely to be younger and female than MUH individuals [53].

Among five of the metabolic health definitions used (excluding the Meigs et al.’s definition [39], which is based solely on HOMA-IR), blood pressure, C-reactive protein, antihypertensive medication use, and HOMA-IR are decisive contributors to the classification of individuals as MUH at baseline. However, regardless of the specific definition or metabolic health phenotype, not all metabolic health parameters included in these definitions show similar improvement between bariatric/metabolic surgery type groups (medication use was not considered, as it is a categorical variable). In fact, besides total, HDL–, and LDL–cholesterol [included in the NCEP ATP III [33,34,35], Karelis et al.’s [37,38], and/or Khan et al.’s [40] definitions], triglycerides [included in the NCEP ATP III [33,34,35], Karelis et al.’s [37,38], and Khan et al.’s [40] definitions] and C-reactive protein [included in the Khan et al.’s [40] and Pluemacher et al.’s definitions] are differently improved within and/or between bariatric/metabolic surgery type groups. Accordingly, these are the definitions that show the highest decrease (above 40%) of MUH percentage from baseline to the first follow-up.

In some publications, with different study designs (in terms of sample size, individuals’ age and gender, metabolic health definition used, type of bariatric/metabolic surgery performed, baseline and post-surgical evaluations, and/or follow-up duration), the impact of bariatric/metabolic surgery upon MH and MUH is compared. Inconsistency exists about which metabolic health phenotype benefits the most from bariatric surgery, with the impact of surgery type not always being evaluated [19,20,21,25,28]. In our study, among the three surgical procedures, RYGB is most frequently associated with a beneficial impact on metabolic features used in each definition and is associated with a higher relative body weight loss. Nevertheless, here, the metabolic health phenotype (1 and 2 y) after surgery does not depend on the type of surgery performed.

Unlike us, Storms et al. [24] reported a progressive increase in total body weight loss and excess BMI loss over the follow-up period, up to 2 y after bariatric surgery. In their study, RYGB (55.6%) and sleeve gastrectomy (44.4%) were performed in 60.8% of females and 58.3% of males, respectively. Their cohort was much smaller than ours (*n* = 133), and all participants were classified as MUH at baseline according to Wildman et al. [60] and van Vliet-Ostaptchouk et al. [48]. Compared to our sample, their population had a similar age (43.10 ± 10.70 vs. 43.24 ± 10.67 y) but differed in gender distribution (lower female proportion: 72.9 vs. 82.7%) and had higher BMI (52.0 ± 8.1 vs. 43.34 ± 5.53 kg/m^2^). Although weight loss was higher with RYGB than with sleeve gastrectomy (similarly to our results), Storms et al. questioned the clinical relevance of this result, as both surgical procedures led to a progressive and sustained weight loss with a trend toward stabilization between 1 and 2 y post-operatively (that we do not observe here–relative body weight loss was similar at those two follow-up time points). In this German study, (a) conversion of MUH-to-MH was independent of surgery type and gender, and (b) triglycerides, glucose, HbA1c, HOMA-IR, hypertension, and C-reactive protein decreased both in absolute values and/or in the percentage of abnormal values; the beneficial increase in HDL–cholesterol absolute values was the exception. A 63% and 53.2% reduction was observed in the number of individuals with abnormal C-reactive protein and HOMA-IR values, respectively, regardless of surgery type or metabolic health phenotype [24]. Overall, when not considering the specific metabolic health definition or phenotype, our study reveals no significant impact of surgery type upon (systolic and diastolic) blood pressure, glucose, HOMA-IR, and glycated hemoglobin absolute values. Regarding the latter, because the Schulze et al.’s definition [2] includes diabetes diagnosis (yes/no) as a variable and since, for the evaluation of the impact of surgery, the parameter needs to be continuous, we analyzed HbA1c as a surrogate. The results show that there is no significant difference because of the type of surgery, but HbA1c values change significantly over time, and this trajectory differs between MH and MUH individuals of the Schulze et al.’s definition [2] (different development across time and always higher in MUH). Regarding C-reactive protein, throughout the study period, a decreasing trend is observed by us in the proportion of MUH individuals with high C-reactive protein values according to Khan et al.’s [40] and Pluemacher et al.’s definitions. This emphasizes the importance of the chosen analytical approach, as trends in subgroups may be obscured when only absolute values are considered and/or when they depend on the specific comparisons made.

Jiménez et al. [25], not distinguishing between RYGB and sleeve gastrectomy, found a significant improvement in the absolute values of all metabolic parameters analyzed in 52 insulin resistant women (43.7 ± 12.0 y; 44.9 ± 3.3 kg/m^2^) 12 months after surgery (namely, blood pressure, glucose, HbA1c, HOMA-IR, lipids, ApoB, high sensitivity C-reactive protein, total leukocyte count, and alanine aminotransferase). Barzin et al. [21], in a cohort of 2244 individuals with morbid obesity (35 ≤ BMI < 40 kg/m^2^; 80.7% female; 18–65 y), reported an increase in excess body weight loss and a decrease in WSC and BMI from 6 to 12 months after bariatric surgery (sleeve gastrectomy or gastric bypass), which stabilized at 24 months, in both metabolic health phenotypes, according to the Joint Interim Statement’s definition [61]. Within MUH, the most relevant abnormal feature (after elevated WSC) was low HDL–cholesterol (78.4%), while the least prevalent metabolic abnormality was elevated blood pressure (57.5%). In our cohort, however, (a) no lipid abnormality is the main determinant of MUH (as anthropometric impairments were considered compulsory for MUH identification); in particular, low HDL–cholesterol is not the most relevant parameter for MUH characterization within the NCEP ATP III [33,34,35], Karelis et al.’s [37,38] or Khan et al.’s [40] definitions, (b) blood pressure is relevant for MUH characterization in NCEP ATP III [33,34,35], Schulze et al.’s [2] and Khan et al.’s [40] definitions, and (c) stabilization of BMI and relative body weight loss also occurs between the first and second y of follow-up. Barzin et al. did not discuss the impact of bariatric/metabolic surgery type (as sleeve gastrectomy and gastric bypass had a similar distribution among metabolic health phenotypes), and so the differential impact of the surgery type upon phenotypes cannot be discussed in this morbid obesity cohort, where a significant improvement in absolute values or percentage of abnormal values of all metabolic parameters analyzed was observed (blood pressure, lipids, and glucose) [21]. Pelascini et al. [20] found that 2 y after RYGB, glycemia, HOMA-IR, HDL–cholesterol, triglycerides, and C-reactive protein significantly improved in MUH.

One limitation of this study is the variability in participant presence across follow-up assessments, with some individuals participating at certain time points but not consistently across all, either due to the existence of missing values (that could not be recovered due to the retrospective nature of the study) or drop-out. This leads to a second limitation: the inability to assess the conversion from MUH to MH over time, which may also be affected by the adaptations made to some definitions during the follow-up period. A third limitation arises from the 11-year recruitment period, which may reflect changes in indications, peri- and post-operative care, as well as medication (although adjustment for calendar year of surgery was considered). Finally, although the sample size is large (*n* = 3313), the study population originates from a single centre and therefore cannot be considered nationally representative.

## 5. Conclusions

Based on our findings, and in a summary and mainly descriptive format, we now revisit and answer the research questions that guided our investigation.

Regarding which metabolic health definition best classifies bariatric/metabolic surgery candidates, at baseline, our results indicate that the two definitions that most consistently identify MH and MUH individuals (Khan et al.’s [40] and NCEP ATP III [33,34,35] definitions) also show, as expected, similar percentages of MUH and a high overlap of the individuals within the MUH classification; however these definitions do not identify the highest proportion of MUH individuals. This would be advantageous if eligibility for bariatric/metabolic surgery were to rely primarily on that phenotype. In this context, we suggest either adopting the Karelis et al.’s definition [37,38], which identifies, at baseline, the largest proportion of MUH in our population, or selecting individuals based on the presence of impaired metabolic features that show the greatest improvement after bariatric/metabolic surgery—namely elevated C-reactive protein, triglycerides, total, and LDL–cholesterol, and low HDL–cholesterol.

This addresses which surgical procedure in our cohort yields the greatest anthropometric and metabolic benefits, and we found that it was RYGB, particularly upon relative body weight loss and total and LDL–cholesterol.

In relation to the definition that most effectively captures post-surgical improvement, from baseline to the first follow-up, we observed that the Khan et al.’s [40], NCEP ATP III [33,34,35], and Karelis et al.’s definitions present similar and high drops in the proportion of MUH. NCEP ATP III [33,34,35] individuals (both MUH and MH) had the smallest relative body weight loss, whereas MH individuals, according to Khan et al. [40], showed the largest relative body weight loss, and MUH individuals, according to Karelis et al. [37,38], showed the third highest, without relevant differences among the three highest groups.

Finally, when examining whether the metabolic features that show the greatest improvement following bariatric/metabolic surgery align with the metabolic health definitions chosen for this study, we found they are criteria mainly for the following definitions: NCEP ATP III [33,34,35] (which includes two of them), Khan et al. [40] (three features), and Karelis et al. [37,38] (four features).

From the revisitation of our research questions within our cohort, the following overarching conclusions can be drawn. Metabolic health phenotypes pre- and post-surgery vary by definition, and the latter are not solely driven by weight loss or procedure type. RYGB shows the greater and/or more frequent beneficial impact upon weight loss and/or metabolic parameters. Our results reinforce the complexity of the metabolic response to bariatric/metabolic surgery and highlight the need for more integrated and rigorous assessments of metabolic health phenotypes.

## Figures and Tables

**Table 1:** Definitions used for metabolic healthy phenotype classification, over time, after bariatric/metabolic surgery.

	NCEP ATP III Modified from 2001, 2002, and 2005 [33,34,35]	Karelis et al. 2004 [37,38]	Meigs et al. 2006 [39]	Khan et al. 2011 [40]	Pluemacher et al. 2024	Schulze et al. 2024 [2]
Glucose and glucose-related parameters	Glucose ≥ 100 mg/dL.			Glucose ≥ 100 mg/dL or use of antidiabetic medication.	Glucose ≥ 100 mg/dL.	No prevalent type 2 diabetes mellitus ^a^.
Insulin resistance		HOMA-IR ≥ 1.95.	HOMA-IR < 75th percentile (among the individuals without diabetes < 6.4).		HOMA-IR ≥ 75th percentile (among the individuals without diabetes ≥ 6.4).	
Blood pressure	SBP/DBP ≥ 130/85 mmHg.			SBP/DBP ≥ 130/85 mmHg or use of antihypertensive medication.	SBP ≥ 130 mmHg.	SBP < 130 mmHg. No use of blood pressure-lowering medication.
Lipid profile	HDL–cholesterol < 40 mg/dL for men or 50 mg/dL for women. Triglycerides ≥ 150 mg/dL.	Triglycerides ≥ 150 mg/dL. Total cholesterol ≥ 200 mg/dL. LDL–cholesterol ≥ 100 mg/mL. HDL–cholesterol ≤ 50 mg/mL.		HDL–cholesterol ≤ 50 mg/dL or use of lipid-lowering medication. Triglycerides ≥ 150 mg/dL.		
Inflammatory markers				CRP ≥ 3.0 mg/dL.	CRP ≥ 3.0 mg/dL.	
**Baseline**						
Metabolically healthy overweight and obesity	≤1 Metabolic abnormality AND WSC > 102 cm for men or 88 cm for women.	≤1 Metabolic abnormality AND BMI ≥ 25 kg/m^2^ OR WSC > 102 cm for men or 88 cm for women.	This criterion AND BMI ≥ 25 kg/m^2^ OR WSC > 102 cm for men or 88 cm for women.	≤2 Metabolic abnormalities AND BMI ≥ 25 kg/m^2^.	≤1 Metabolic abnormalities AND BMI ≥ 25 kg/m^2^.	All these features AND waist-to-hip ratio < 0.95 for women and < 1.03 for men.
**Follow-up**						
Metabolically healthy normal weight: BMI < 25 kg/m^2^ AND WSC ≤ 102 cm for men or 88 cm for women	≤2 Metabolic abnormalities.	≤1 Metabolic abnormality.	This criterion (HOMA-IR < 2 for 1st–3rd follow-ups and <2.2 for the 4th follow-up).	≤2 Metabolic abnormalities.	≤1 Metabolic abnormalities (HOMA-IR ≥ 2 for 1st–3rd follow-ups and ≥2.2 for the 4th follow-up).	All these features.
** Metabolically healthy overweight: BMI 25–29.9 kg/m^2^ AND WSC ≤ 102 cm for men or 88 cm for women. ** Metabolically healthy obesity: BMI ≥ 30 kg/m^2^ or WSC > 102 cm for men or 88 cm for women	≤1 Metabolic abnormality.	≤1 Metabolic abnormality.	This criterion (HOMA-IR < 2 for 1st–3rd follow-ups and <2.2 for the 4th follow-up).	≤2 Metabolic abnormalities.	≤1 Metabolic abnormalities (HOMA-IR ≥ 2 for 1st–3rd follow-ups and <2.2 for the 4th follow-up).	All these features.

BMI, body mass index; CRP, C-reactive protein; DBP, diastolic blood pressure; HDL, high-density lipoprotein; HOMA-IR, Homeostatic Model Assessment for Insulin Resistance; LDL, low-density lipoprotein; SBP, systolic blood pressure; WSC, waist circumference. ** Keeps in consideration the overweight, obesity and overweight + obesity groups described in the Methods section, besides the metabolic abnormality number. ^a^ Defined according to the American Diabetes Association [43].

**Table 2:** Age period for the start of increased weight according to the metabolic health phenotype at baseline.

Metabolic Health Phenotype Definition	Baseline Metabolic Health Phenotype	Start of Overweight or Obesity *n* (%)			*p*-Value
		Childhood (<10 y)	Adolescence (10–20 y)	Adult Age (>20 y)	
NCEP ATP III, modified from 2001, 2002, and 2005 [33,34,35]	MH	249 (38.9)	114 (41.2)	285 (34.6)	0.049
	MUH	376 (60.2)	163 (58.8)	539 (65.4)	
	OR	Ref	1.006 [0.752; 1.347]	1.022 [0.814; 1.284]	
Karelis et al. 2004 [37,38]	MH	91 (13.9)	35 (11.2)	76 (8.7)	0.006
	MUH	564 (86.1)	277 (88.8)	797 (91.3)	
	OR	Ref	1.283 [0.846; 1.946]	1.668 [1.193; 2.333]	
Meigs et al. 2006 [39]	MH	498 (72.1)	241 (73.0)	653 (69.9)	0.461
	MUH	193 (27.9)	89 (27.0)	281 (30.1)	
	OR	Ref	0.958 [0.714; 1.287]	1.088 [0.869; 1.363]	
Khan et al. 2011 [40]	MH	232 (34.9)	120 (38.3)	291 (30.6)	0.024
	MUH	432 (65.1)	193 (61.7)	660 (69.4)	
	OR	Ref	0.928 [0.698; 1.234]	0.934 [0.746; 1.169]	
Pluemacher et al. 2024	MH	91 (20.5)	41 (20.7)	113 (19.3)	0.846
	MUH	353 (79.5)	157 (79.3)	474 (80.7)	
	OR	Ref	1.030 [0.679; 1.563]	0.935 [0.676; 1.292]	
Schulze et al. 2024 [2]	MH	122 (18.9)	66 (22.1)	117 (13.1)	<0.001
	MUH	522 (81.1)	233 (77.9)	775 (86.9)	
	OR	Ref	0.878 [0.621; 1.241]	1.001 [0.740; 1.353]	

MH, metabolically healthy phenotype; MUH, metabolically unhealthy phenotype; Ref, reference; Chi-square test. OR (odds ratio) adjusted to age at surgery.

**Table 3:** Relative body weight loss over time according to the metabolic health phenotype at baseline.

Metabolic Health Phenotype Definition	Baseline Phenotype Group	Relative Body Weight Loss (%) (Mean ± SD)			
		1st Follow-Up	2nd Follow-Up	3rd Follow-Up	4th Follow-Up
NCEP ATP III, modified from 2001, 2002, and 2005 [33,34,35]	MH	31.30 ± 10.48	31.09 ± 11.49	28.98 ± 11.60 ^#^	26.89 ± 11.74 ^###; &&&^
	MUH	31.71 ± 9.83	31.47 ± 10.41	29.52 ± 10.84 ^###^	27.45 ± 10.63 ^###; &&&^
Karelis et al. 2004 [37,38]	MH	33.85 ± 9.55	33.26 ± 10.39	31.67 ± 10.40 ^a^	29.27 ± 12.61 ^#; c^
	MUH	32.66 ± 9.79	32.53 ± 10.48	29.83 ± 11.39 ^###^	28.17 ± 10.91 ^###; &&&^
Meigs et al. 2006 [39]	MH	32.84 ± 9.83	32.83 ± 10.55	30.52 ± 11.25 ^###^	28.97 ± 11.31 ^###; &&&^
	MUH	32.56 ± 9.52	32.29 ± 9.91	29.49 ± 10.75 ^###^	27.38 ± 10.03 ^###; &&^
Khan et al. 2011 [40]	MH	34.66 ± 8.83	34.91 ± 8.98	32.54 ± 9.96 ^##^	30.32 ± 10.93 ^###; &&&^
	MUH	33.23 ± 8.66 ***	33.17 ± 9.35 ***	31.38 ± 9.70 ^###, b^	29.17 ± 9.83 ^###; &&&^
Pluemacher et al. 2024	MH	33.44 ± 9.30	33.53 ± 8.86	31.11 ± 9.82	28.55 ± 12.40 ^&^
	MUH	33.86 ± 8.46	33.75 ± 9.13	31.35 ± 9.81 ^###^	29.02 ± 9.80 ^###; &&&^
Schulze et al. 2024 [2]	MH	32.33 ± 10.07	32.36 ± 10.48	29.98 ± 11.16 ^#^	28.29 ± 10.83 ^##; &&^
	MUH	31.76 ± 9.78	31.61 ± 10.74	29.81 ± 10.82 ^###^	27.67 ± 10.99 ^###; &&&^

Relative body weight loss: (initial body weight–body weight at each time point) × 100/initial body weight. After bariatric/metabolic surgery, follow-up lasted up to four years (one follow-up per year, and the first follow-up was one year after the surgery). MH, metabolically healthy phenotype; MUH, metabolically unhealthy phenotype; SD, standard deviation. Comparisons of MH vs. MHU within each definition were evaluated by an unpaired Student *t*-test; *** *p* < 0.005, ^b^ = 0.096. Comparisons along time, for a given phenotype, within each definition, were evaluated by paired Student *t*-test; third/fourth vs. first follow-up ^#^ *p* < 0.05, ^##^ *p* < 0.005, ^###^ *p* < 0.001, ^a^ = 0.072 while fourth vs. third follow-up ^&^ *p* < 0.05, ^&&^ *p* < 0.01, ^&&&^ *p* < 0.001, ^c^ = 0.05.

**Table 4:** Number of altered metabolic features in the metabolically unhealthy phenotype over the follow-up period.

Evaluation Time Points	Weight Groups	Number of Metabolic Features in Metabolically Unhealthy Phenotype (Mean ± SD)				
		NCEP ATP III Modified from 2001, 2002, and 2005 [33,34,35]	Karelis et al. 2004 [37,38]	Khan et al. 2011 [40]	Pluemacher et al. 2024	Schulze et al. 2024 [2]
Baseline	OW + OB	2.56 ± 0.686	3.08 ± 0.921	3.69 ± 0.720	2.71 ± 0.761	1.79 ± 0.842
1st Follow-up	OW + OB	2.20 ± 0.477 *	2.54 ± 0.755 *	3.37 ± 0.585 *	2.44 ± 0.595 *	1.31 ± 0.479 *
	OW	2.04 ± 0.196	2.31 ± 0.550	3.17 ± 0.380	2.31 ± 0.503	1.26 ± 0.441
	OB	2.26 ± 0.535	2.66 ± 0.816	3.42 ± 0.623	2.47 ± 0.613	1.33 ± 0.494
	NW	3.00 ± 0.000	2.13 ± 0.409	3.29 ± 0.469	2.24 ± 0.436	1.07 ± 0.361
2nd Follow-up	OW + OB	2.26 ± 0.549 *	2.48 ± 0.691 *	3.32 ± 0.531 *	2.45 ± 0.600 *	1.33 ± 0.510 *
	OB	2.29 ± 0.588	2.57 ± 0.743	3.37 ± 0.564	2.45 ± 0.597	1.38 ± 0.532
	OW	2.12 ± 0.326	2.30 ± 0.539	3.14 ± 0.351	2.44 ± 0.619	1.19 ± 0.420
	NW	3.00± 0.000	2.20 ± 0.467	3.13 ± 0.354	2.14 ± 0.363	1.07 ± 0.264
3rd Follow-up	OW + OB	2.25 ± 0.515 *	2.51 ± 0.751 *	3.34 ± 0.579 *	2.49 ± 0.644 *	1.37 ± 0.512 *
	OB	2.27 ± 0.517	2.59 ± 0.790	3.36 ± 0.592	2.55 ± 0.661	1.41 ± 0.531
	OW	2.19 ± 0.512	2.36 ± 0.641	3.21 ± 0.528	2.28 ± 0.528	1.27 ± 0.446
	NW	3.00 ± 0.000	2.20 ± 0.401	3.33 ± 0.516	2.38 ± 0.518	1.17 ± 0.388
4th Follow-up	OW + OB	2.27 ± 0.520 *	2.54 ± 0.684 *	3.33 ± 0.531 *	2.51 ± 0.649 *	1.35 ± 0.547 *
	OW	2.14 ± 0.378	2.33 ± 0.492	3.00 ± 0.000	2.10 ± 0.316	1.09 ± 0.292
	OB	2.29 ± 0.536	2.64 ± 0.736	3.41 ± 0.564	2.57 ± 0.664	1.41 ± 0.577
	NW	3.00 ± 0.000	2.32 ± 0.548	3.00 ± 0.000	2.20 ± 0.447	1.33 ± 0.500

NW, normal weight; OB, obesity; OW, overweight; OW + OB, overweight + obesity; SD, standard deviation. After bariatric/metabolic surgery, follow-up lasted up to four years (one follow-up per year, and the first follow-up was one year after the surgery). Meigs et al.’s 2006 [39] definition was not included in this analysis because it only considers one feature for the metabolic health classification. Comparisons between baseline and each follow-up for OW + OB: * *p* < 0.05; unpaired Student’s *t*-test.

**Table 5:** Relative body weight loss differences by type of surgery and follow-up period.

		Compared Against	RBWL Mean Difference (%)	95% Confidence-Interval (%)	*p*-Value
1st Follow-up	RYGB	Sleeve gastrectomy	2.53	[1.70; 3.37]	<0.001
		Gastric band	17.89	[16.47; 19.31]	<0.001
	Sleeve gastrectomy	Gastric band	15.35	[13.86; 16.85]	<0.001
2nd Follow-up	RYGB	Sleeve gastrectomy	4.37	[3.39; 5.35]	<0.001
		Gastric band	18.76	[16.51; 20.53]	<0.001
	Sleeve gastrectomy	Gastric band	14.39	[12.70; 16.08]	<0.001
3rd Follow-up	RYGB	Sleeve gastrectomy	4.02	[2.83; 5.22]	<0.001
		Gastric band	16.87	[14.83; 18.90]	<0.001
	Sleeve gastrectomy	Gastric band	12.84	[10.89; 14.79]	<0.001
4th Follow-up	RYGB	Sleeve gastrectomy	4.78	[3.31; 6.25]	<0.001
		Gastric band	15.59	[13.37; 17.81]	<0.001
	Sleeve gastrectomy	Gastric band	10.81	[8.44; 13.17]	<0.001

RYGB, Roux-en-Y gastric bypass; RBWL, relative body weight loss. After bariatric/metabolic surgery, follow-up lasted up to four years (one follow-up per year, and the first follow-up was one year after the surgery). One-way ANOVA with Tukey post hoc test.

**Table 6:** Diabetes progression by surgery type.

	Gastric Band, *n* (%)		RYGB, *n* (%)		Sleeve Gastrectomy, *n* (%)	
	**Diabetes at Baseline**					
	No	Yes	No	Yes	No	Yes
**Diabetes at 1st follow-up**						
No	195 (99%)	16 (67%)	1325 (100%)	137 (95%)	687 (99%)	51 (88%)
Yes	1 (1%)	8 (33%)	2 (0%)	8 (5%)	4 (1%)	7 (12%)
	**Diabetes at 1st follow-up**					
	No	Yes	No	Yes	No	Yes
**Diabetes at 2nd follow-up**						
No	138 (97%)	3 (50%)	1057 (100%)	3 (43%)	516 (99%)	4 (40%)
Yes	4 (3%)	3 (50%)	3 (0%)	4 (57%)	5 (1%)	6 (60%)
	**Diabetes at 2nd follow-up**					
	No	Yes	No	Yes	No	Yes
**Diabetes at 3rd follow-up**						
No	101 (96%)	2 (50%)	767 (99%)	4 (67%)	372 (99%)	2 (33%)
Yes	4 (4%)	2 (50%)	6 (1%)	2 (33%)	5 (1%)	4 (67%)
	**Diabetes at 3rd follow-up**					
	No	Yes	No	Yes	No	Yes
**Diabetes at 4th follow-up**						
No	73 (96%)	2 (40%)	548 (99%)	3 (38%)	288 (99%)	2 (25%)
Yes	3 (4%)	3 (60%)	4 (1%)	5 (62%)	3 (1%)	6 (75%)

RYGB, Roux-en-Y gastric bypass.

**Table 7:** High blood pressure progression by surgery type.

	Gastric Band, *n* (%)		RYGB, *n* (%)		Sleeve Gastrectomy, *n* (%)	
	**High Blood Pressure at Baseline**					
	No	Yes	No	Yes	No	Yes
**High blood pressure at 1st follow-up**						
No	16 (52%)	31 (44%)	120 (77%)	242 (61%)	46 (75%)	87 (55%)
Yes	15 (48%)	39 (56%)	36 (23%)	154 (39%)	15 (25%)	72 (45%)
	**High blood pressure at 1st follow-up**					
	No	Yes	No	Yes	No	Yes
**High blood pressure at 2nd follow-up**						
No	18 (75%)	10 (29%)	151 (80%)	33 (36%)	51 (81%)	15 (31%)
Yes	6 (25%)	24 (71%)	38 (20%)	60 (64%)	12 (19%)	33 (69%)
	**High blood pressure at 2nd follow-up**					
	No	Yes	No	Yes	No	Yes
**High blood pressure at 3rd follow-up**						
No	15 (83%)	6 (32%)	69 (71%)	22 (35%)	27 (69%)	7 (23%)
Yes	3 (17%)	13 (68%)	28 (29%)	40 (65%)	12 (31%)	24 (77%)
	**High blood pressure at 3rd follow-up**					
	No	Yes	No	Yes	No	Yes
**High blood pressure at 4th follow-up**						
No	9 (64%)	4 (40%)	51 (77%)	21 (43%)	8 (73%)	9 (56%)
Yes	5 (36%)	6 (60%)	15 (23%)	28 (57%)	3 (27%)	7 (44%)

RYGB, Roux-en-Y gastric bypass.

## Data Availability

The original contributions presented in this study are included in the article/Supplementary Material. Further inquiries can be directed to the corresponding authors.

## Supplementary Materials

The following supporting information can be downloaded at: https://www.mdpi.com/article/10.3390/metabo16010047/s1, Figure S1A,B shows how many individuals were eligible, how many had each outcome available at each time point, and how this looks by procedure; Table S1 discloses the individuals detailed baseline metabolic characterization; Table S2 presents the body mass index absolute values and body mass index groups; Table S3 shows the type of surgery according to the metabolic health phenotype at baseline; Table S4 reveals the impact of the type of surgery upon metabolic parameters and relative body weight loss; Table S5 presents the metabolic health classification at follow-ups one and two according to the type of surgery.
