# Supplementary material for: Metabolic Outcomes in Bariatric/Metabolic Surgery Individuals: Impact of Metabolic Health Definition, Type of Surgery, and Follow-Up Duration—An Observational, Retrospective Study
Source: Metabolites. 2026 Jan 5;16(1):47. doi: 10.3390/metabo16010047 (PMC12843696; doi:10.3390/metabo16010047)
Supplement: Supplementary file 1 [file metabolites-16-00047-s001.zip › metabolites-4028670-supplementary.pdf]

Metabolic Outcomes in Bariatric/Metabolic Surgery Individuals: Impact of Metabolic Health Definition, Type of Surgery, and Follow-Up Duration—An Observational, Retrospective Study

Anna Pluemacher [1,2]; Cláudia Camila Dias [3,4]; Bárbara Peleteiro [5,6,7,8]; Denise Pinheiro [2]; Paula Freitas [9,10,11]; Eduardo Lima [9,11]; Alexandra Leitão [12,13]; Elisabete Martins [13,14,15]; Maria João Martins [2,10,*]

[1] Faculty of Nutritional Sciences, University of Potsdam, 14476 Potsdam, Germany
[2] Unit of Biochemistry, Department of Biomedicine, Faculty of Medicine, University of Porto, 4200-319 Porto, Portugal
[3] Knowledge Management Unit, Faculty of Medicine, University of Porto, 4200-319 Porto, Portugal
[4] RISE-Health, Department of Community Medicine, Information and Health Decision Sciences (MEDCIDS), Faculty of Medicine, University of Porto, 4200-319 Porto, Portugal
[5] Centro de Epidemiologia Hospitalar, Unidade Local de Saúde São João, 4200-319 Porto, Portugal
[6] Departamento de Ciências da Saúde Pública e Forenses e Educação Médica, Faculdade de Medicina, Universidade do Porto, 4200-319 Porto, Portugal
[7] EPIUnit, Instituto de Saúde Pública, Universidade do Porto, 4050-600 Porto, Portugal
[8] Laboratório para a Investigação Integrativa e Translacional em Saúde Populacional (ITR), Universidade do Porto, 4050-600 Porto, Portugal
[9] Faculdade de Medicina, Universidade do Porto, 4200-319 Porto, Portugal
[10] Instituto de Investigação e Inovação em Saúde (i3S), Universidade do Porto, 4200-135 Porto, Portugal
[11] Integrated Responsibility Center for Obesity (CRI-O), São João Local Health Unit, 4200-319 Porto, Portugal
[12] Departamento de Medicina Interna, Unidade Local de Saúde de Barcelos/Esposende, 4754-909 Barcelos, Portugal
[13] RISE-Health, Faculty of Medicine, University of Porto, 4200-319 Porto, Portugal
[14] Serviço de Cardiologia, ULS S. João EPE, 4200-319 Porto, Portugal
[15] Department of Medicine, Faculty of Medicine, University of Porto, 4200-319 Porto, Portugal
* Correspondence: mmartins@med.up.pt

Supplementary Tables S1 to S5.

Supplementary Table S1. Individuals detailed baseline metabolic characterization.

	P25	Median	P75
**Age y**	36	43	51
**BMI (kg/m²)**	39.56	42.60	46.20
**Waist circ (cm)**	114.0	121.6	131.0
**Hip circ (cm)**	124.0	131.0	138.0
**WtHR**	0.87	0.93	0.99
**SBP (mmHg)**	124	136	148
**DBP (mmHg)**	80	82	91
**HR (bpm)**	73	80	89
**Hemoglobin (g/dL)**	12.90	13.60	14.40
**AST (U/L)**	18.0	22.0	28.0
**ALT (U/L)**	18	24	36
**gGT (U/L)**	20.0	27.0	42.0
**ALP (U/L)**	63	75	91
**TC (mg/dL)**	168	192	218
**HDL-C (mg/dL)**	42	48	56
**LDL-C (mg/dL)**	96.0	118.0	140.0
**Triglycerides (mg/dL)**	89	119	162
**Glucose (mg/dL)**	86	95	109
**HbA1c (%)**	5.3	5.6	6.0
**HOMA-IR**[1]	2.83	4.41	6.92
**Insulin (µU/mL)**	12.20	18.30	26.60
**Creatinine (mg/dL)**	0.58	0.67	0.79
**Uric acid (mg/dL)**	4.60	5.40	6.30
**CRP (mg/L)**	4.80	8.55	14.10
**Vitamin D (ng/mL)**	11.00	15.00	21.00
**Albumin (g/L)**	39.6	41.4	43.3
**Albuminuria (mg/L)**	3.70	6.20	13.80

ALP, alkaline phosphatase; ALT, Alanine aminotransferase; AST, aspartate aminotransferase; BMI, body mass index; circ, circumference; CRP, c-reactive protein; DBP, diastolic blood pressure; HbA1c, glycated haemoglobin; HDL-C, HDL-cholesterol; HOMA-IR, Homeostatic Model Assessment for Insulin Resistance; HR, heart rate; gGT, g-glutamyl transferase; LDL-C, LDL-cholesterol; SBP, systolic blood pressure; SD, standard deviation; TC, total cholesterol; WtHR, waist-to-hip ratio. Regarding normality, the variables diastolic blood pressure, hip circumference, heart rate, and albumin showed a normal distribution according to the Shapiro test.

[1] Calculated according to Matthews, D.R.; Hosker, J.P.; Rudenski, A.S.; Naylor, B.A.; Treacher, D.F.; Turner, R.C. Homeostasis model assessment: insulin resistance and beta-cell function from fasting plasma glucose and insulin concentrations in man. *Diabetologia* **1985**; 28:412-419.

Supplementary Table S2. Body mass index absolute values and body mass index groups.

Body mass index			Body mass index groups			
**Evaluation time**	**n**	**Median (Min; Max) (kg/m²)**	**Normal weight% (n)**	**Overweight% (n)**	**Obese% (n)**	**Overweight + Obese% (n)**
**All individuals**						
**Baseline**	3313	42.60 (24.46; 75.78)				100 (3313)
**1st Follow-up**	2771	28.72 (19.33; 56.81)	18.29 (507)	34.05 (944)	47.66 (1321)	81.7 (2265)
**2nd Follow-up**	2280	28.44 (18.74; 55.96)	17.4 (397)	35.3 (804)	47.3 (1079)	82.6 (1883)
**3rd Follow-up**	1812	29.33 (18.73; 57.26)	14.1 (255)	35.3 (640)	50.6 (917)	85.9 (1557)
**4th Follow-up**	1229	30.33 (19.05; 59.09)	10.5 (129)	32.8 (404)	56.7 (698)	89.5 (1102)
**NCEP ATP III individuals** [33-35]						
**Baseline**	1791	42.59 (29.90; 73.64)				100 (1791)
**1st Follow-up**	1203	29.00 (19.36; 51.10)	15.7 (189)	30.8 (371)	53.4 (643)	84.3 (1014)
**2nd Follow-up**	811	28.63 (19.35; 50.13)	15.9 (129)	30.5 (247)	53.6 (435)	84.1 (682)
**3rd Follow-up**	551	29.57 (19.82; 50.60)	12.5 (69)	29.4 (162)	58.1 (320)	87.3 (482)
**4th Follow-up**	332	30.55 (22.23; 55.06)	8.4 (28)	24.7 (82)	66.9 (222)	91.6 (304)
**Karelis et al. individuals** [37, 38]						
**Baseline**	1955	42.08 (24.46; 73.64)				100 (1955)
**1st Follow-up**	2170	28.67 (19.33; 56.81)	18.4 (400)	33.8 (734)	47.7 (1036)	81.6 (1770)
**2nd Follow-up**	1626	28.28 (18.74; 55.96)	17.7 (287)	36.5 (593)	45.9 (746)	82.3 (1339)
**3rd Follow-up**	1237	29.11 (18.73; 53.95)	14.2 (176)	36.5 (452)	49.2 (609)	85.8 (1061)
**4th Follow-up**	833	29.90 (19.05; 59.09)	11.6 (97)	34.3 (286)	54.0 (450)	88.4 (736)
**Meigs et al. individuals** [39]						
**Baseline**	2079	42.17 (24.46; 73.64)				100 (2079)
**1st Follow-up**	2183	28.67 (19.33; 56.81)	18.5 (403)	33.9 (739)	47.7 (1041)	81.5 (1780)
**2nd Follow-up**	1643	28.28 (18.74; 55.96)	17.9 (294)	36.2 (594)	46.0 (755)	82.1 (1349)
**3rd Follow-up**	1253	29.10 (18.73; 53.95)	14.4 (180)	36.5 (457)	49.2 (616)	85.6 (1073)
**4th Follow-up**	842	29.90 (19.05; 59.09)	11.5 (97)	34.4 (290)	54.0 (455)	88.5 (745)
**Khan et al. individuals** [40]						
**Baseline**	2035	42.10 (28.36; 73.64)				100 (2035)
**1st Follow-up**	1983	28.55 (19.33; 56.81)	19.3 (382)	34.2 (678)	46.5 (923)	80.7 (1601)
**2nd Follow-up**	1504	28.15 (18.74; 55.96)	18.5 (278)	36.2 (545)	45.3 (681)	81.5 (1226)
**3rd Follow-up**	1106	29.17 (18.73; 53.95)	14.4 (159)	35.3 (390)	50.4 (557)	85.6 (947)
**4th Follow-up**	764	30.02 (19.05; 55.06)	11.3 (86)	33.6 (257)	55.1 (421)	88.7 (678)
**Pluemacher et al. individuals**						
**Baseline**	1278	41.65 (29.90; 73.64)				100 (1278)
**1st Follow-up**	1068	28.97 (19.36; 51.10)	16.1 (172)	30.9 (330)	53.0 (566)	83.9 (896)
**2nd Follow-up**	716	28.51 (19.35; 50.13)	15.9 (114)	30.2 (216)	53.9 (386)	84.1 (602)
**3rd Follow-up**	465	29.38 (19.82; 50.60)	14.4 (67)	28.8 (134)	56.8 (264)	85.6 (398)
**4th Follow-up**	280	30.42 (22.23; 55.06)	9.3 (26)	24.3 (68)	66.4 (186)	90.7 (254)
**Schulze et al. individuals** [2]						
**Baseline**	1902	42.70 (27.36; 73.64)				100 (1902)
**1st Follow-up**	1156	28.96 (19.36; 51.10)	15.9 (184)	31.3 (362)	52.8 (610)	84.1 (972)
**2nd Follow-up**	811	28.73 (19.35; 50.13)	15.9 (129)	30.3 (246)	53.8 (436)	84.1 (682)
**3rd Follow-up**	545	29.39 (20.42; 50.60)	12.1 (66)	31.0 (169)	56.9 (310)	87.9 (479)
**4th Follow-up**	339	30.45 (21.68; 55.06)	8.8 (30)	25.4 (86)	65.8 (223)	91.2 (309)

Supplementary Table S3. Type of surgery according to the metabolic health phenotype at baseline.

Metabolic health phenotype definition	Baseline metabolic health phenotype	Type of surgery, n (%)			*p*-value
		RYGB	Sleeve gastrectomy	Gastric Band	
**NCEP ATP III, modified from 2001, 2002 & 2005 [33-35]**	**MH**	418 (37.4)	158 (33.3)	91 (47.2)	0.004
	**MUH**	699 (62.6)	316 (66.7)	102 (52.8)	
**Karelis et al. 2004 [37, 38]**	**MH**	141 (11.4)	64 (10.6)	12 (10.9)	0.879
	**MUH**	1094 (88.6)	538 (89.4)	98 (89.1)	
**Meigs et al. 2006 [39]**	**MH**	929 (70.9)	450 (69.2)	85 (77.3)	0.223
	**MUH**	381 (29.1)	200 (30.8)	25 (22.7)	
**Khan et al. 2011 [40]**	**MH**	431 (33.1)	237 (33.4)	5 (31.3)	0.977
	**MUH**	870 (66.9)	472 (66.6)	11 (68.8)	
**Pluemacher et al. 2024**	**MH**	157 (19.3)	91 (20.2)	2 (28.6)	0.779
	**MUH**	657 (80.7)	360 (79.8)	5 (71.4)	
**Schulze et al. 2024 [2]**	**MH**	194 (16.3)	87 (16.6)	29 (16.0)	0.980
	**MUH**	996 (83.7)	437 (83.4)	152 (84.0)	

MH, metabolically healthy phenotype; MUH, metabolically unhealthy phenotype; RYGB, Roux-en-Y gastric bypass; Chi-square test.

Supplementary Table S4. Impact of the type of surgery upon metabolic parameters and relative body weight loss.

Parameter	Surgery type		Mean difference	95% Confidence-Interval	*p*-value
Triglycerides (mg/dL)	Gastric band	RYGB	11.515	[4.334; 18.697]	< 0.001
		Sleeve gastrectomy	11.611	[4.029;19.194]	< 0.001
	RYGB	Sleeve gastrectomy	0.095	[-4.120;4.311]	> 0.999
Total Cholesterol (mg/dL)	Gastric band	RYGB	18.078	[13.009;23.147]	< 0.001
		Sleeve gastrectomy	8.525	[3.171;13.879]	< 0.001
	RYGB	Sleeve gastrectomy	-9.553	[-12.520; -6.587]	< 0.001
LDL-Cholesterol (mg/dL)	Gastric band	RYGB	18.475	[14.321; 22.629]	< 0.001
		Sleeve gastrectomy	9.540	[5.150; 13.930]	< 0.001
	RYGB	Sleeve gastrectomy	-8.936	[-11.384; -6.486]	< 0.001
HDL-Cholesterol (mg/dL)	Gastric band	RYGB	-1.814	[-3.467;- 0.161]	0.026
		Sleeve gastrectomy	-2.660	[-4.407;- 0.915]	< 0.001
	RYGB	Sleeve gastrectomy	-0.847	[-1.816; 0.123]	0.110
HbA1c (%)	Gastric band	RYGB	0.019	[-0.553; 0.592]	> 0.999
		Sleeve gastrectomy	0.136	[-0.469; 0.741]	> 0.999
	RYGB	Sleeve gastrectomy	0.116	[-0.222; 0.455	> 0.999
Systolic blood pressure (mm Hg)	Gastric band	RYGB	1.007	[-1.201; 3.216]	0.824
		Sleeve gastrectomy	0.786	[-1.576; 3.147]	> 0.999
	RYGB	Sleeve gastrectomy	-0.222	[-1.631; 1.188]	> 0.999
Diastolic blood pressure (mm Hg)	Gastric band	RYGB	1.110	[-0.370;2.590]	0.217
		Sleeve gastrectomy	0.972	[-0.610; 2.555]	0.423
	RYGB	Sleeve gastrectomy	-0.138	[-1.082; 0.807]	> 0.999
HOMA-IR[1]	Gastric band	RYGB	0.394	[-0.138; 0.926]	0.228
		Sleeve gastrectomy	0.395	[-0.163; 0.952]	0.271
	RYGB	Sleeve gastrectomy	-0.001	[-0.298; 0.299]	> 0.999
Glucose (mg/dL)	Gastric band	RYGB	1.015	[-1.987; 4.018]	> 0.999
		Sleeve gastrectomy	1.629	[-1.545; 4.804]	0.657
	RYGB	Sleeve gastrectomy	0.614	[-1.167; 2.395]	> 0.999
C-Reactive protein (mg/L)	Gastric band	RYGB	2.439	[1.129; 3.749]	< 0.001
		Sleeve gastrectomy	2.235	[0.872; 3.597]	< 0.001
	RYGB	Sleeve gastrectomy	-0.204	[-0.890; 0.482]	> 0.999
Relative body weight loss (%)	Gastric band	RYGB	-16.835	[-18.312; -15.358]	< 0.001
		Sleeve gastrectomy	-14.085	[-15.644; -12.527]	< 0.001
	RYGB	Sleeve gastrectomy	2.750	[1.867; 3.633]	< 0.001

HbA1c, glycated haemoglobin; HOMA-IR, Homeostatic Model Assessment for Insulin Resistance; RYGB, Roux-en-Y gastric bypass; linear mixed-effects models, adjusted for age, gender, BMI at baseline, type of surgery, and calendar year of surgery. [1] Calculated according to Matthews, D.R.; Hosker, J.P.; Rudenski, A.S.; Naylor, B.A.; Treacher, D.F.; Turner, R.C. Homeostasis model assessment: insulin resistance and beta-cell function from fasting plasma glucose and insulin concentrations in man. *Diabetologia* **1985**; 28:412-419.

Supplementary Table S5. Metabolic health classification at follow-ups one and two according to the type of surgery.

Type of surgery	Follow-up	Metabolically unhealthy phenotype, in the OW + OB n (%)					
		NCEP ATP III, modified from 2001, 2002, and 2005 [33-35]	Karelis et al. 2004 [37, 38]	Meigs et al. 2006 [39]	Khan et al. 2011 [40]	Pluemacher et al. 2024	Schulze et al. 2024 [2]
RYGB	1st	91 (14.9)	348 (33.8)	221 (21.3)	127 (13.2)	117 (21.4)	301 (50.4)
	2nd	61 (15.6)	263 (33.8)	174 (22.2)	75 (10.4)	69 (20.5)	187 (48.3)
	p-value	0.960	> 0.999	0.897	0.214	0.949	0.813
Sleeve gastrectomy	1st	52 (19.0)	323 (59.3)	172 (31.4)	87 (17.6)	88 (36.5)	153 (57.1)
	2nd	43 (21.5)	248 (56.6)	147 (33.3)	72 (18.1)	65 (35.5)	114 (56.7)
	p-value	0.795	0.705	0.832	0.976	0.978	0.997
Gastric band	1st	40 (30.8)	144 (73.8)	121 (62.1)	62 (44.3)	77 (72.0)	69 (65.1)
	2nd	25 (27.8)	88 (72.1)	72 (58.5)	40 (38.5)	48 (59.3)	63 (67.7)
	p-value	0.892	0.945	0.823	0.659	0.188	0.925

RYGB, Roux-en-Y gastric bypass; OB, obesity; OW, overweight; Chi-square test.

Supplementary Figure S1.

A.

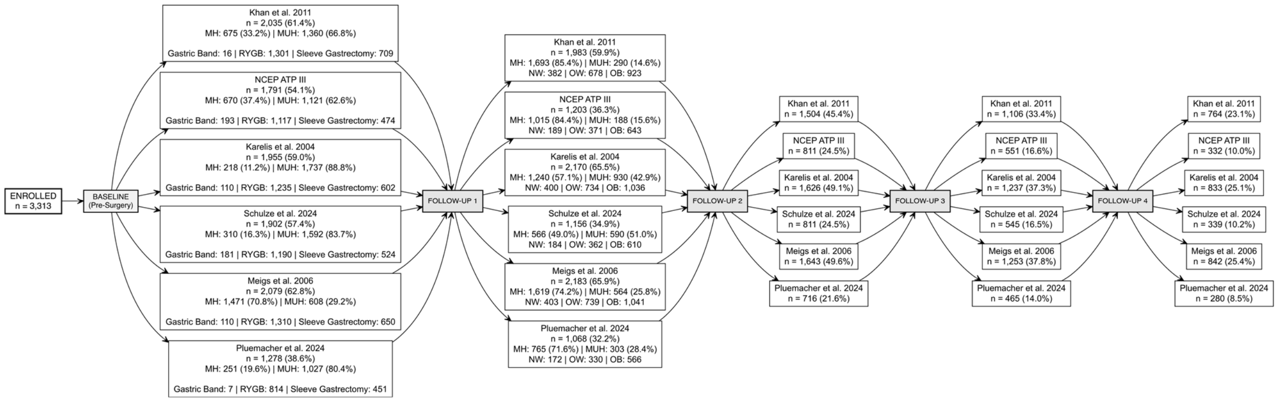

B.

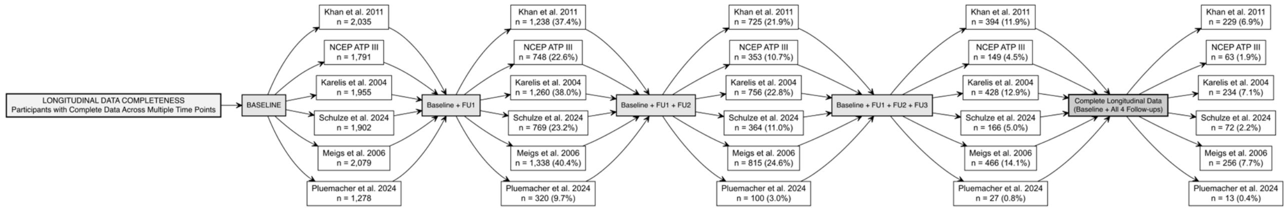

Supplementary Figure 1 shows how many individuals were eligible, how many had each outcome available at each time point, and how this looks by procedure.

Panel A shows the complete participant flow across all time points (baseline plus all four follow-ups) for each of the six metabolic health definitions. It displays how many individuals had complete data at each time point, broken down by metabolic health *status* and weight groups (the combined group, overweight + obesity, is not included, because it is simply the sum of the overweight and obesity groups). At baseline, the distribution of surgery types for each definition is also provided to give full transparency about sample composition.

Panel B shows longitudinal data completeness, illustrating how many individuals had complete data across cumulative time points (e.g., baseline + follow-up 1, baseline + follow-up 2 + follow-up 3, etc.). The final row shows participants with complete data at all five time points (baseline plus all four follow-ups).

FU, follow-up; NW, normal-weight; OB, obesity group; OW, overweight group; RYGB, Roux-en-Y gastric bypass.
